# Asymmetric Catalysis: Recent Advances toward a More Sustainable Synthesis

**DOI:** 10.1021/acscentsci.5c01874

**Published:** 2026-02-09

**Authors:** Tanguy Saget, Mohamed Mellah, Philippe Dauban, Emmanuelle Schulz

**Affiliations:** † Université Paris-Saclay, CNRS, Institut de Chimie des Substances Naturelles, UPR 2301, 91198 Gif-sur-Yvette, France; ‡ Institut de Chimie Moléculaire et des Matériaux d’Orsay, UMR 8182, Université Paris-Saclay, CNRS, Bâtiment Henri Moissan, 91400 Orsay, France

## Abstract

Asymmetric catalysis is the most sustainable means of achieving efficient production of enantioenriched compounds, which are valuable to the pharmaceutical industry and advanced functionalized materials. The field has undergone considerable development since its inception about 60 years ago. The development of new chiral ligands or organocatalysts or the discovery of new reactions all led to spectacular progress in terms of activity and enantioselectivity. In this context, this Outlook article aims to highlight major advances that demonstrate the central role of asymmetric catalysis in sustainability, with the examples discussed representing a selection shaped by the authors’ scientific perspective and sensitivity. This includes recent innovations in catalyst recycling, the use of less precious and less toxic metals in asymmetric organometallic catalysis, as well as the control of the positioning of catalytic sites, to further improve their efficiency. The development of asymmetric electrocatalysis and photocatalysis as well as of mechanochemistry and continuous flow chemistry is also discussed. Finally, the complexity and step-economy issues are raised with the presentation of relevant examples of asymmetric multicatalytic processes and new challenging transformations that include late-stage functionalization reactions, molecular edition or reactions targeting the creation of axial, planar or helicoidal chirality.

## Graphical abstract

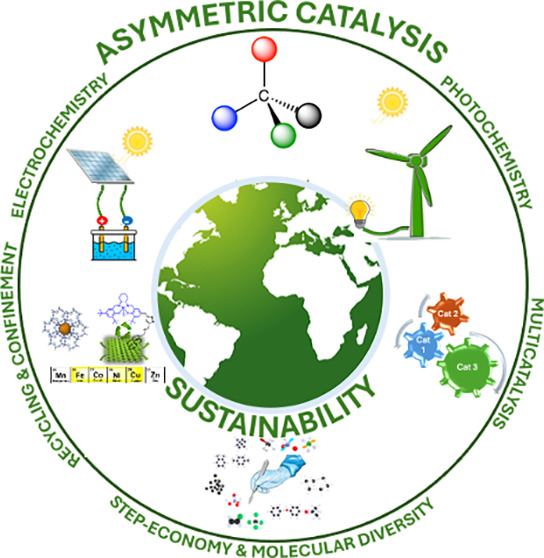

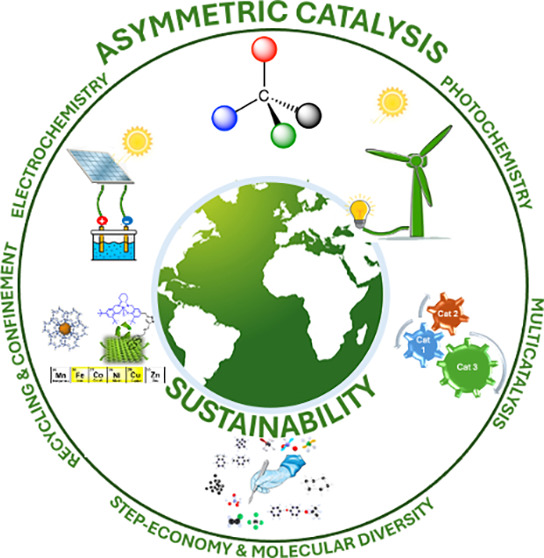

## 1 Introduction

Chirality lies at the heart of life sciences. The four major classes of biomolecules, i.e., proteins, nucleic acids, carbohydrates and lipids, are chiral. This structural property plays a major role in their mutual interactions, which are the foundation of communication in complex biological systems. Thus, the design of pharmaceuticals aimed at interfering with biomolecules often relies on chiral molecules.^1^ This is clearly demonstrated by the Figure 1, which highlights that 58% (256) of the small molecules approved by the FDA and the EMA between 2013 and 2022 (442) are chiral enantiopure products.^2^ It is worth mentioning that 10 additional drugs (less than 3% of the small molecule drugs) were commercialized as chiral racemic compounds in the same time frame. The same holds true for agrochemicals with more than 40% of the approved products from 2007 to 2023 being chiral enantiopure compounds.^3^

**Figure 1:**
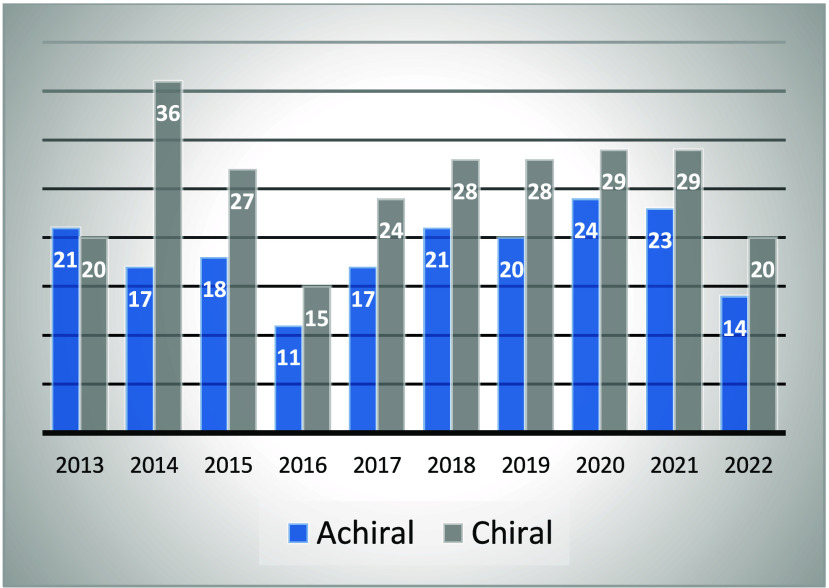
Comparison of the number of achiral (blue) and chiral enantiopure (gray) small molecules approved by the FDA and the EMA from 2013 to 2022.

Chirality is also of paramount importance in material sciences. It is well acknowledged that the stereochemistry has a crucial influence on the crystallinity of polymers and, thus, on their mechanical properties as well as their stability.^4^ In addition, growing attention is paid to the light-matter interaction with outstanding achievements having been reported in optoelectronics through the design of chiral conjugated systems interacting with circular polarized light.^5^ Accordingly, the ubiquity of chirality testifies to the still relevant importance of asymmetric synthesis, and more particularly of asymmetric catalysis in the context of sustainable chemistry.

Asymmetric catalysis can be defined as a process in which a chiral catalyst directs the formation of a new element of chirality in a molecule so that the resulting stereoisomeric products are formed in different amounts. The first example of a metal-catalyzed asymmetric reaction was reported by the Nozaki and Noyori group. It dates back to 1966 with the catalytic cyclopropanation of styrene and homologation of 2-phenyloxetane in the presence of a chiral Schiff base-copper­(II) complex. Both transformations afford the corresponding products of carbene addition with an enantiocontrol evaluated by the optical rotation.^6^ Numerous achievements based on transition metal catalysis were then reported in the following decades, among which the rhodium-catalyzed asymmetric alkene hydrogenation pioneered by Knowles and Kagan^7,8^ as well as the Sharpless asymmetric epoxidation^9^ stand out. The impact of asymmetric catalysis on science was then first acknowledged by the Nobel Prize awarded in 2001 to Sharpless, Noyori and Knowles for their seminal contributions to the field.^10–12^

It is often believed that the pioneering work of Noyori on copper-catalyzed cyclopropanation corresponds to the birth of asymmetric catalysis, however, the first example of a catalytic enantioselective process was reported earlier in 1960 with the alkaloid-catalyzed asymmetric synthesis of α-phenylpropionates from ketenes.^13^ Then, seminal papers on amino acid-catalyzed asymmetric Strecker reaction and Robinson annulation appeared in the early 1970s.^14,15^ But it was not until the 2000s that organocatalysis really expanded,^16^ as it was considered ineffective or limited in terms of applications and substrates scope. Thus, significant breakthroughs were made in 2000 with the papers simultaneously published by List and MacMillan, respectively on the proline-catalyzed intermolecular aldol reaction,^17^ and amine-catalyzed cycloadditions.^18^ Since then, organic catalysis has become a major focus that was also combined with other type of catalytic processes, notably metal catalysis and photocatalysis, to achieve even greater diversity. It represents a significant step toward the development of sustainable methods, which in turn was recognized with the Nobel Prize awarded in 2021 to List and MacMillan.

Biocatalysis is the third pillar of asymmetric catalysis, perfectly complementing organometallic and organic catalysis. Seminal studies in this area were reported over a century ago,^19^ and the field has expanded considerably in the beginning of the 21st century with the application of molecular biology to the production of engineered enzymes using a directed evolution strategy.^20^ This approach has enabled the development of a biocatalytic toolbox that can be applied to various reactions, even those unknown in nature, and to total syntheses based on multienzymatic sequences. The concept of the directed evolution of enzymes, which was also acknowledged with the 2018 Nobel Prize awarded to Arnold, is a burgeoning domain, however, it will be only briefly discussed in this Outlook as it lies out of its scope.

The concept of catalysis is undoubtedly associated with that of sustainability, as the ninth principle of green chemistry, and its connection to waste minimization and energy saving.^21^ The progress made in the design of transition metal- and organo-catalyzed asymmetric transformations have been invaluable to this end. Nevertheless, limitations, typically high catalyst loadings or the use of precious metals, still remain. Moreover, the conceptualization of the green chemistry introduced by Anastas and Werner in 1998 ^21^ has made organic chemists aware of the need to adopt a comprehensive approach to design more efficient processes. Thus, since the 2001 Nobel Prize, numerous strategies have been implemented to address the issue of the impact of synthetic chemistry on the environment. In this Outlook, we put forward a focused and opinionated overview of recent achievements in asymmetric catalysis that highlight its strong interconnections with sustainable chemistry, grounded in the authors’ expertise and perspective.

## 2 RECYCLING, CONFINEMENT AND USE OF ABUNDANT METALS

The development of enantioselective catalysis initially remained closely linked to the discovery of Wilkinson’s rhodium catalyst and its stabilization by chiral ligands for the enantioselective hydrogenation of increasingly sophisticated substrates. The fact remains that, due to its scarcity and therefore its high cost, rhodium is not a metal aligned with sustainable development, despite its very high efficiency in producing nearly enantiopure substrates even at low catalytic loadings.^22^ Nevertheless, it should be noted that shortly after the fundamental discoveries in asymmetric catalysis, industrial processes were developed and efficiently exploited. Thus, the Monsanto process using a chiral rhodium-dipamp complex enabled the preparation of a precursor of L-DOPA used in the treatment of Parkinson’s disease, by asymmetric hydrogenation.^23^ Nevertheless, at the same time, the cyclopropanation reaction with chiral copper complexes enabled the preparation of cyclopropane carboxylic acids of industrial interest (Sumitomo Chemical Co).^24^ This context led researchers, from the earliest days of development, to devise strategies for easy recovery and reuse of these precious species in order to improve their productivity. Thus, homogeneous supported catalysis was practically born at the same time as its homogeneous version. Kagan imagined to anchor the catalyst on a solid support, keeping the advantages of the catalysis in solution, and he used a commercially available chlorinated Merrifield resin, after oxidation, to attach a Rh­(I)-diop complex.^25^ However, this first system showed a much lower efficiency than its homogeneous counterpart but these preliminary results were very important in assessing the effect of the support on the catalytic behavior of the metal complex. The Stille group therefore proposed the use of a better-suited support by preparing a cross-linked polymer containing methacrylate-based monomers modified with the same Rh­(I)-diop complex.^26^ This way, the authors succeeded in the enantioselective hydrogenation of acylamidoacrylic acids with efficiencies matching those obtained under homogeneous conditions, albeit with a lower reaction rate.

The same strategy of immobilization for recycling purposes was also successfully applied to organocatalysis^27,28^ to mitigate the use of high catalytic loadings^29^ or recover complex and valuable catalysts,^30^ and some processes were even developed on a large scale for the synthesis of drugs.^31^

Consequently, the search for efficient and recyclable chiral catalysts has increased considerably with the quest for a detailed understanding of the role of the support, which must go beyond simple heterogenization for easy recovery. Various types of immobilization strategies were explored and these advances are described in detail in comprehensive reviews.^32^ Numerous examples can be found for asymmetric metal-catalyzed hydrogenation reactions due to the historical context, so the following examples will focus on other type of processes, operating sometimes with high catalytic loadings, and for which catalyst recovery and reuse is very desirable.

The search for efficient and recyclable chiral catalysts has increased considerably with the quest for a detailed understanding of the role of the support, which must go beyond simple heterogenization for easy recovery.

When seeking efficient heterogeneous catalysis, the primary goal remains to achieve efficiency values, both in terms of activity and selectivity, that match or surpass those achieved under homogeneous conditions. To this purpose, the support structure and the nature of the linker are key parameters. To give just one very recent example as illustration, Hang, Zhu and their group reported the easy preparation of nickel nanoparticles embedded in silica in the presence of a (*N*,*N*)-diphenylethylene-diamine-based chiral ligand.^33^ This supported catalyst was tested to promote the asymmetric Michael addition of dimethyl malonate to nitroalkenes, demonstrating superior activity compared to its homogeneous counterpart, calculated with a 3.6-fold higher apparent turnover frequency, for small nanoparticles (Scheme 1). Nonlinear effect studies, and analyses of the supported catalyst (STEM, BET, XPS) led the authors to propose that the peripheral Ni sites at the interface formed by contact between the Ni NPs and the silica play an important positive role in the course of the reaction. Catalyst stability was also evaluated in both batch and flow, but the ligand had to be added continuously to obtain stable activity and selectivity values, indicating its poor coordination with the support, while almost no Ni leaching could be measured.

**Scheme 1:**
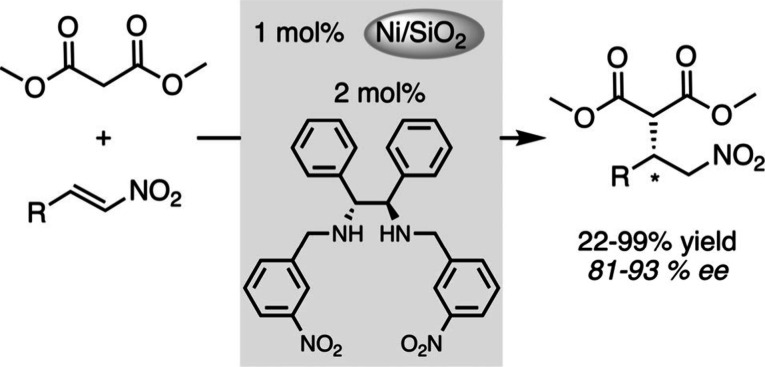
Asymmetric Michael Addition Catalyzed by Supported Ni NPs with Chiral Diamine

However, the pursuit of consistent recycling efficiency is also a key driver in the development of new processes for asymmetric heterogeneous catalysis.^34^ This task is difficult to achieve, particularly in fundamental studies, where the quantities of catalyst used are always very small and where, as a result, it is difficult to implement effective recycling procedures, especially in batch mode. Traditionally, recycling attempts are carried out over 3 to 5 cycles, with few reaching a dozen reuses. Such number was achieved by Gu, Tu and You in the context of asymmetric heterogeneous dearomatization reactions, thanks to the heterogenization of complex chiral phosphoric acids (CPA) as organocatalysts. CPAs are highly effective but difficult and costly to synthesize, and they often require a fairly high catalytic ratio.^35^ The authors therefore prepared chiral microporous polymer networks through Friedel–Crafts reactions between phosphoric acid derivatives functionalized with anthracenyl groups and biphenyldibenzyl alcohol, with the anthracene groups being essential for creating a sterically hindered environment and a highly cross-linked system (Scheme 2). These species proved highly active and selective at a 5 mol % ratio, to promote the asymmetric transfer hydrogenation of various 2-phenylquinolines with Hantzsch ester and the asymmetric dearomative amination of β-naphthols with azodicarboxylate. In both cases, the solid porous organocatalysts were recovered by centrifugation and then reused in a new catalysis, without any loss of activity or enantioselectivity for 10 additional cycles.

**Scheme 2:**
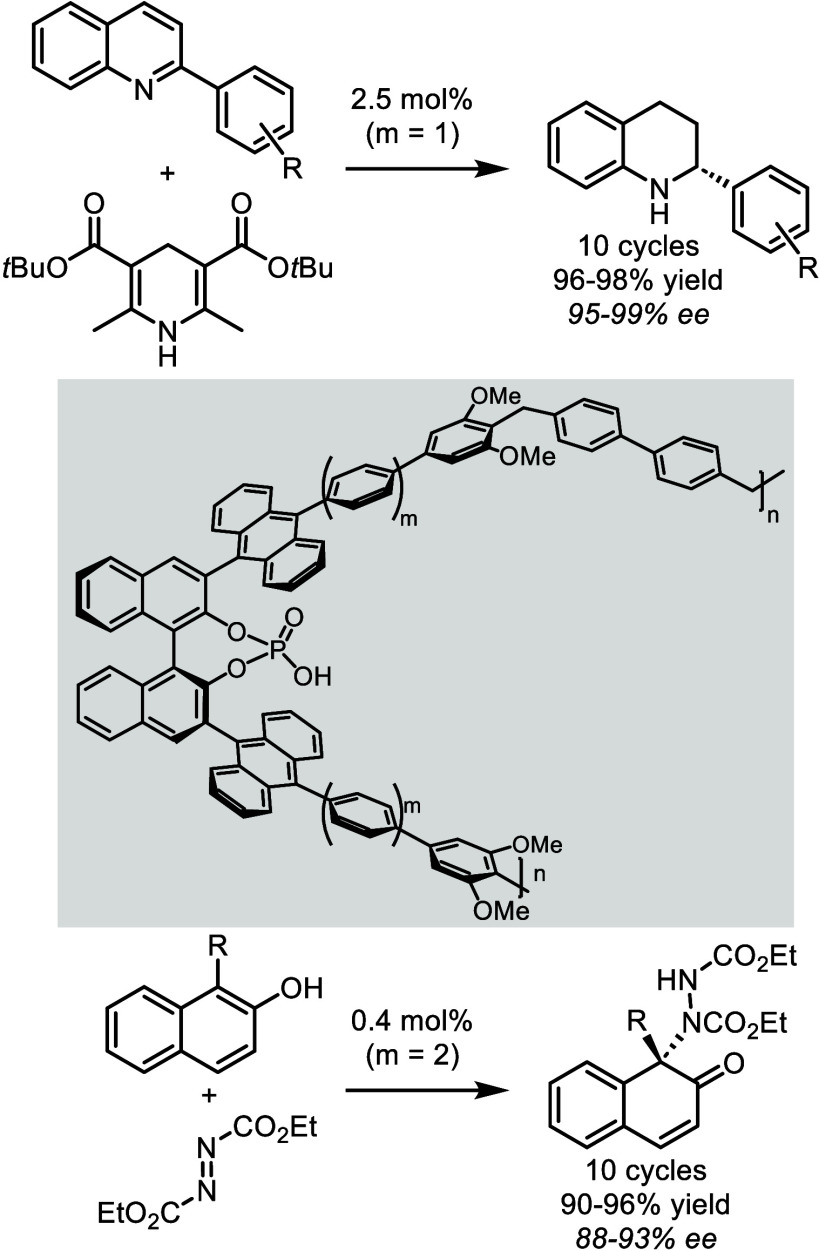
Chiral Phosphoric Acid-Catalyzed Dearomatization

Beyond stable and numerous recycling cycles, immobilizing chiral catalysts can improve their performance, with enantioselectivity values in target products exceeding those obtained under homogeneous conditions. This can occur, for instance, when the heterogenization process is aimed at controlling the positioning of catalytic sites relative to each other, either by increasing the distance between them or bringing them closer together as required. Thus, a process for immobilizing manganese complexes used in oxidation reactions has made it possible to prevent their decomposition, which can easily occur in the homogeneous phase through the formation of inactive dimeric μ-oxo species. Toward this end, graphene oxide (GO) sheets were modified by catalytic active sites through covalent bonds, owing to the presence of oxygenated functional groups on their basal planes and edges. Tan, Yin et al. then covalently grafted imidazole fragments onto GO sheets, which were quaternized by immobilizing a Mn-salen complex (Scheme 3a) that was used for the epoxidation of a wide range of nonfunctionalized olefins in the presence of aqueous NaOCl.^36^ The catalyst immobilized on GO enabled the epoxidation of olefins at a high reaction rate, while also offering high enantioselectivity values. Recovered after centrifugation, it was reused seven times and maintained high yield and enantioselectivity for styrene epoxide, thus demonstrating high stability. It is interesting to note that the authors used the same strategy to fix Ti-salen complexes on GO, arguing that multiple anchoring to the surface could promote cooperative bimetallic catalysis, which is of paramount importance for their test reaction, namely the asymmetric oxidation of sulfides with aqueous H_2_O_2_ (Scheme 3b). It enabled the efficient and selective oxidation of methylaryl sulfides, with the support promoting the formation of di-μ-oxo Ti-salen species, which are true catalytic precursors.^37^ According to the amount of catalyst loading (either the Mn-salen or the Ti-salen), two catalytic oxidation reactions could be achieved. The first one requires site-isolation whereas the other one relies on site-cooperativity, which combines efficiencies superior to those obtained with homogeneous analogues and enables the recycling of catalytic species.

**Scheme 3:**
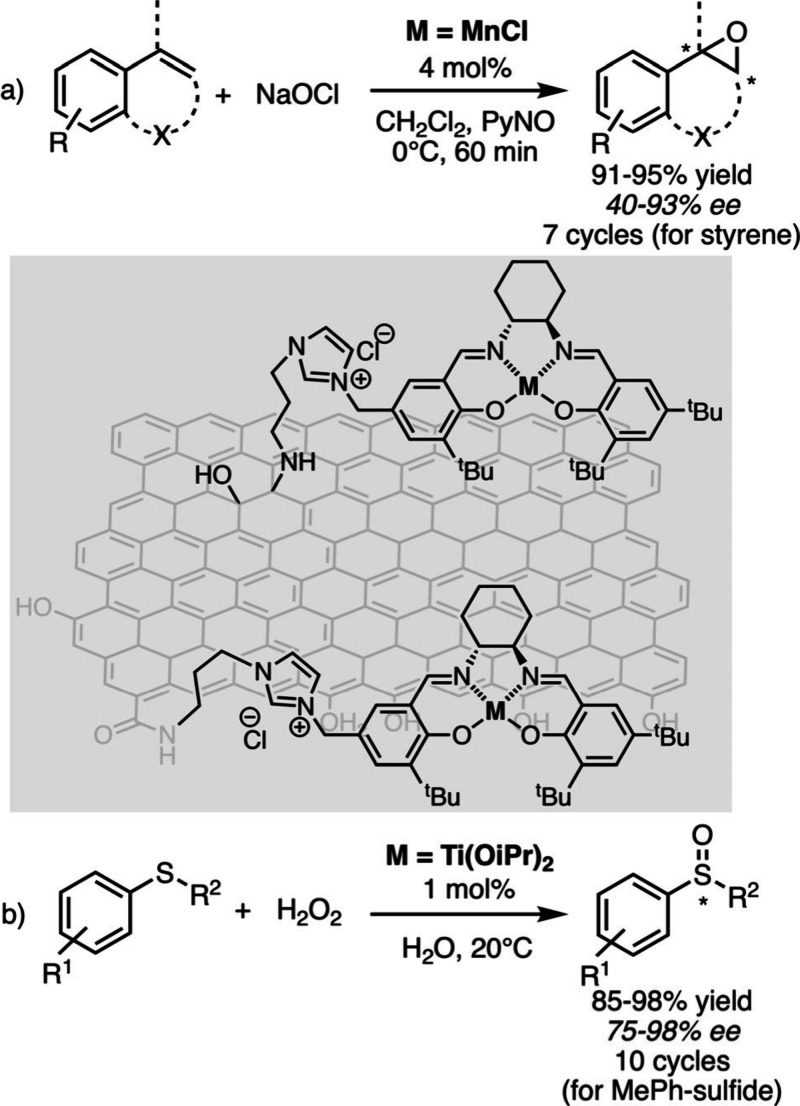
Oxidation Reactions Promoted by a) Mn- and b) Ti-Salen Complexes Immobilized on Graphene Oxide

Another benefit of grafting catalysts onto a support is the possibility of positioning two active catalytic sites close together. This is often challenging in homogeneous catalysis, where it typically requires the complex synthesis of a bifunctional catalyst. A relevant example was described by Jacobsen and his group for the hydrolytic kinetic resolution (HKR) of epoxides. The mechanism of this important transformation was elucidated by Jacobsen and Blackmond and involves a bimetallic cooperative activation of both the nucleophile and the electrophile by salen-cobalt active sites.^38^ In this context, salen ligands were functionalized with a long alkyl chain bearing a terminal thiol group and were further immobilized on gold colloids before incorporation of the active cobalt metal.^39^ Used in the HKR of hexane-1-oxide, the supported catalyst demonstrated high selectivity and accelerated reaction rates compared to its homogeneous counterpart, revealing effective cooperativity made possible by the proximity of the catalytic sites (Scheme 4a). After a simple filtration, the catalyst was used effectively up to seven times before its activity decreased significantly. A reactivation step in the presence of triflic acid was then necessary to restore its activity. Some of us have described the preparation of insoluble cobalt catalysts that can be reused several times by avoiding this reactivation step. Indeed, the electrolytic conditions for the anodic oxidative generation of insoluble salen-cobalt polymers have been precisely adjusted to allow formation of (Co-OAc) and (Co-BF_4_) active sites inside the same polymer structure. The formers are transformed into (Co-OH) species by water activation, while the Lewis-acidic and non-nucleophilic (Co-BF_4_) sites essentially activate the epoxide. This specificity allows the polymer to retain its activity during recycling, even once all (Co-OAc) sites have been hydrolyzed and without the need for additional acidic reactivation (Scheme 4b).^40^

**Scheme 4:**
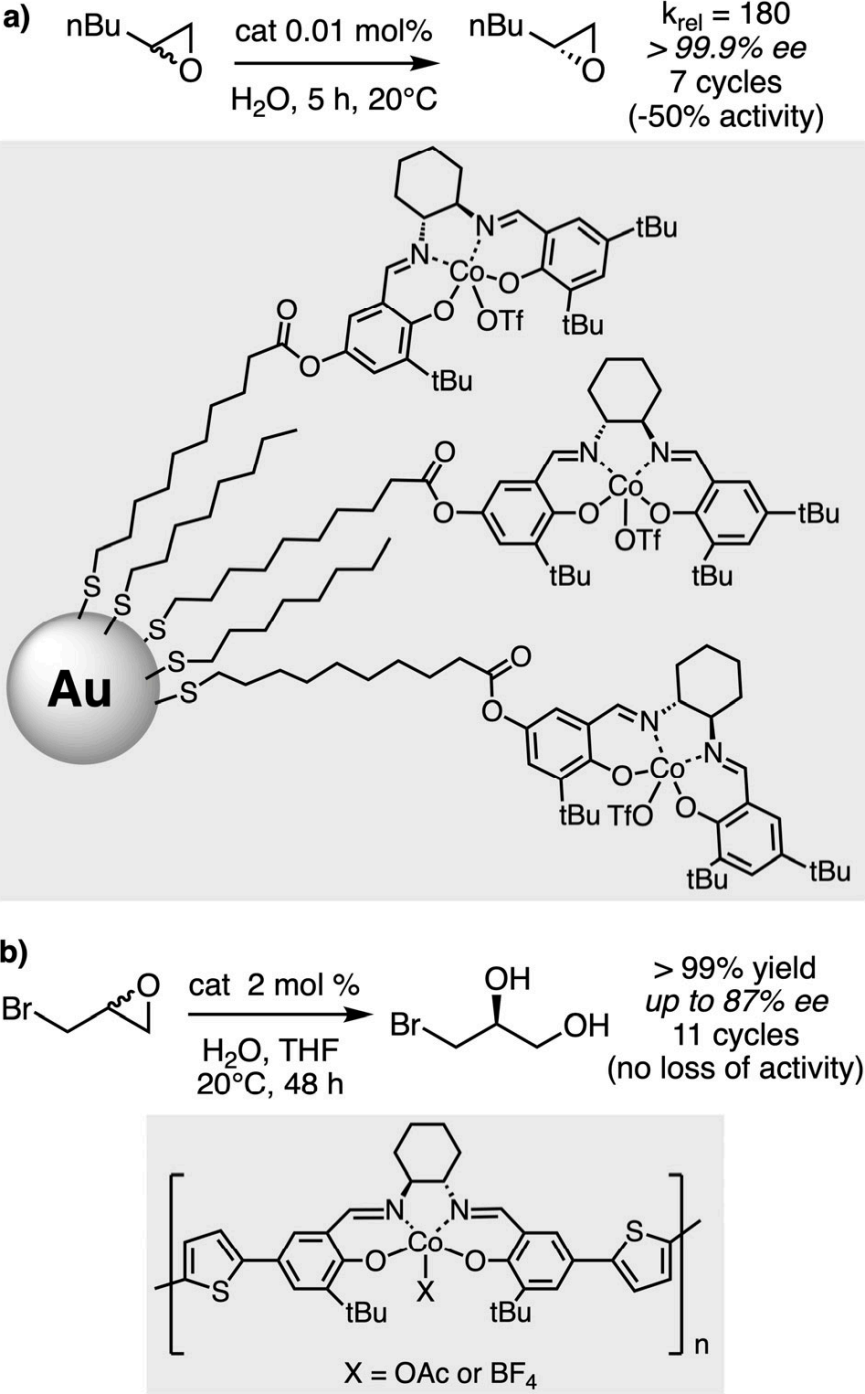
Hydrolytic Kinetic Resolution of Terminal Epoxides by Co-Salen Complexes a) Immobilized on Gold Nanoparticles or B) Electropolymerized

The initial goal of recovering chiral catalysts can thus enhance not only their durability through easy reuse, but also their efficiency via innovative immobilization techniques. Another important step toward sustainability concerns the use of metals that are more abundant in the Earth’s crust and less toxic than the Rh, Ru, and Ir complexes.^41^ Some examples employing Ti, Mn or Co have already been described above, and efforts are currently being made to use abundant copper, a metals that was restricted for a long time to Lewis acidic activation mode.^42^ Recently, the generation of chiral copper-hydride led to efficient enantioselective hydroamination reactions^43,44^ and enantioselective hydrogenation of carbonyl compounds^45^ with very high efficiency. Even some dynamic kinetic resolution processes leading to the simultaneous formation of two contiguous stereogenic centers could be achieved with excellent diastereoselectivity and enantioselectivity.^46^ Very recently, chiral copper hydrides also proved to be remarkable for promoting the kinetic resolution of prochiral 2*H*-azirine-2-carboxylates, leading to valuable azirine and aziridine derivatives with high enantioselectivity values (Scheme 5).^47^

**Scheme 5:**
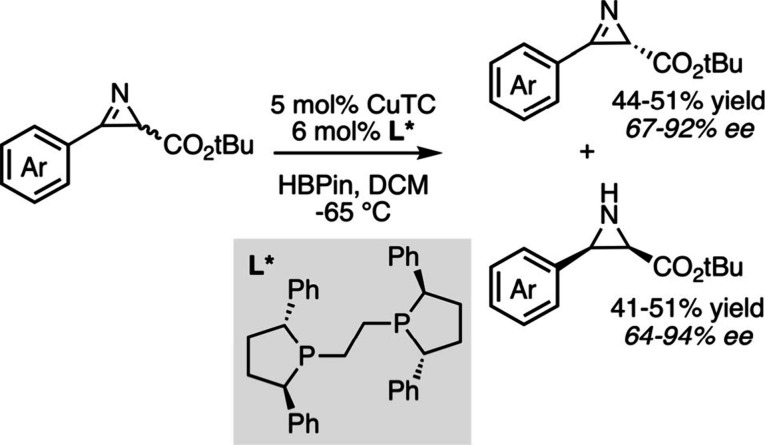
Copper-Catalyzed Reductive Kinetic Resolution of 2*H*-Azirine-2-carboxylates

However, the metal that remains indisputably in number one position for green chemistry is still iron, not only because of its availability and low price but also because of its poor toxicity. In addition, iron has many accessible oxidation states and therefore offers a variety of activation modes. When associated with chiral ligands, iron complexes can promote a wide range of asymmetric catalytic reactions.^48^ A very recent example illustrating the Lewis acid ability of chiral iron salts, is the inverse-electron-demand [4 + 2] cycloaddition/cheletropic retro-[4 + 1] extrusion of SO_2_ reactions between thiophene *S*,*S*-dioxides and 3-substituted indoles to access exahydrocarbazoles bearing a quaternary stereogenic center. This methodology was effectively applied to the total synthesis of (+)-geissoschizoline, an indole alkaloid with various biological activities.^49^ Significant research has also been conducted to develop efficient asymmetric hydrogenation and transfer hydrogenation catalyzed by iron. It relies on in-depth mechanistic studies combined with the development of specific tetradentate chiral ligands bearing tertiary phosphine and secondary amine donor groups, enabling the formation of a bifunctional H–Fe–NH unit in the catalytic cycle.^50^ The transfer of dihydrogen to the polar bonds of ketones or imines occurred readily to produce the corresponding alcohols or amines with excellent yields and enantioselectivity values exceeding 90% in most cases. Meggers and his group expanded the use of iron complexes in asymmetric catalysis by designing chiral-at-iron catalysts bearing achiral *N*-(2-pyridyl)-substituted *N*-heterocyclic carbene bidentate ligands, whose helical arrangement provided metal-centered Λ or Δ*c*onfiguration (Scheme 6). Even if access to these structures is not straightforward, the complexes proved configurationally stable after chemical resolution and could successfully be tested as catalysts for the enantioselective intramolecular Cannizzaro reaction demonstrating the efficient use of a stereogenic iron center.^51^

**Scheme 6:**
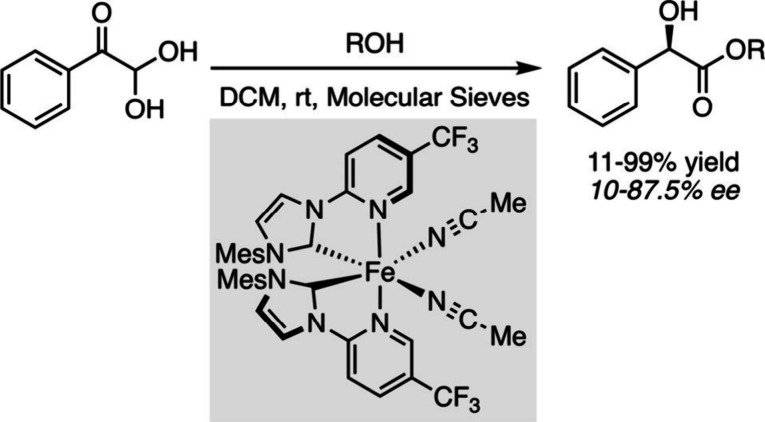
Chiral-at-Iron-Catalyzed Enantioselective Intramolecular Cannizzaro Reaction

In addition to the development of new catalysts, it is also important to highlight the growing interest in creating specific catalytic networks designed to precisely position confined catalytic sites, with the goal of enhancing both their selectivity and durability. Major advances have indeed been made with the development of metal organic frameworks (MOFs), that are stable, porous, and well-defined structures, primarily designed for gas storage and separation.^52^ The activity of these ordered networks stems either from unsaturated metal coordination sites and/or from active chiral linkers between metals, as initially discovered and exploited by Lin’s group. They prepared an enantiopure binaphthol ligand modified with pyridine, this functionality being positioned so as to connect the Cd atoms forming a noninterpenetrating 3D network with very large chiral channels. After adding Ti­(O*i*Pr)_4_, the resulting chiral heterogeneous catalyst proved to be highly effective in promoting enantioselective additions of ZnEt_2_ to aromatic aldehydes, with complete conversion and enantioselectivity values comparable to those obtained from the homogeneous counterpart.^53^ This first example which did not describe recycling studies, has inspired numerous research projects over the past 25 years, with a particular focus on sustainability.^54^ For example, the preparation of a chiral MOF from Cr-salen complexes and CdI_2_, capable of promoting four different catalytic reactions, demonstrated impressive versatility. The same catalytic structure could indeed be used to obtain functionalized cyclopentenones through the Nazarov reaction of alkoxydienones, to carry out the asymmetric aminolysis of *trans*-stilbene oxide, the Diels–Alder and the hetero Diels–Alder reactions. In some cases, a higher level of enantiocontrol can be achieved when compared to that observed under homogeneous conditions, thereby proving the existence of favorable confinement effects and ideal site localization (Scheme 7).^55^ The heterogeneous catalyst was reused in four cycles, indicating good stability.

**Scheme 7:**
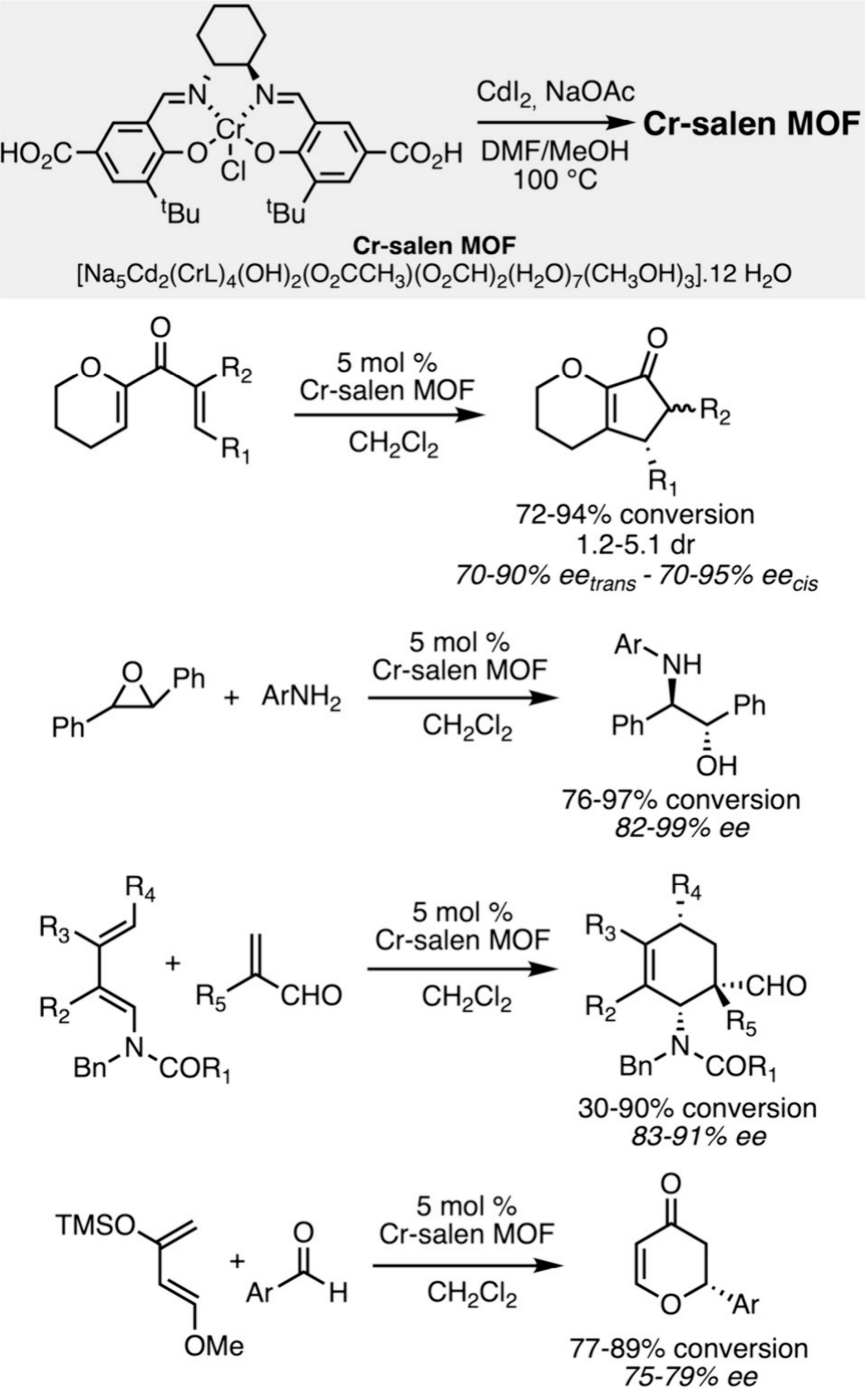
Various Asymmetric Catalytic Reactions Promoted by the Same Cr-Salen MOF

The manipulation of these coordination networks is perfectly mastered in order to modulate their structure, for example through postmodification. There are many impressive examples in the field of metal-salen MOF derivatives with synthetic modifications made directly to the salen ligand inserted into the network strut to modulate its activity,^56^ or through solvent-assisted linker exchange to introduce other asymmetric catalysts into the crystalline solid, opening the door to cooperative^57^ or tandem asymmetric catalysis.^58^ These networks have recently confirmed their usefulness for immobilizing chiral organocatalysts, namely imidophosphates (IDP), which only lead to high enantioselectivity values for homogeneous asymmetric catalysis in their *O*,*O*-syn conformation.^59^ The incorporation of IDP into Dy-MOFs, which leads to the stabilization of the catalytic sites in their active conformation, has enabled significant advances in the asymmetric *O*,*O*-acetalization of diols and aldehydes, while also providing good reusability for the heterogeneous catalyst.

In addition to networks prepared by coordination bonds, covalent-organic frameworks (COFs) have also recently emerged as molecular platforms for providing new crystalline and heterogeneous chiral catalysts that mimic stable nanoreactors for asymmetric catalysis.^60^ Bimetallic and even trimetallic COFs have been prepared from salen-based ligands and have demonstrated excellent activity, enantioselectivity and recyclability in various transformations, such as the cyanation of aldehydes (V@COFs), the Diels–Alder reaction (Co@COFs), the epoxidation of chromene derivatives (Fe@ or Mn@COFs), or the aminolysis of stilbene oxide (Cr@COFs).^61^ The use of these stable networks has recently been extended to include electrochemical asymmetric catalysis. The preparation of a 2D chiral COF, incorporating a thiophene-type organic conducting linker and a MacMillan imidazolinone catalyst, yielded a stable and recyclable material for the electrochemical-induced asymmetric α-arylation of aldehydes.^62^

Recent advances in organometallic and asymmetric organic catalysis have moved beyond simple recycling and metal recovery, focusing instead on improving catalyst efficiency through precise control of site positioning. It is noteworthy that the use of nonprecious metals is rapidly expanding, further broadening the range of applications for these reactions. Future advances are already underway, and they no longer involve only organometallic or organic catalysis alone. The aim is to avoid co-oxidants or coreductants, to use flow techniques and grinding processes, or to carry out combined multicatalysis processes, with the primary objective of saving atoms, solvents, and energy. This is described in the following chapters but remains subject to promising future developments.

## 3 CATALYTIC ASYMMETRIC METHODS POWERED BY RENEWABLE ENERGIES AND NEW PROCESSES

The growing interest in synthetic methods like photochemistry and electrochemistry, particularly when powered by renewable energy, stems from urgent environmental challenges.

These approaches aim to support the long-term and sustainable development of the energy system and to contribute to the transition to a zero-emission society.^63^ This dynamic is driven by standardized setups, which have facilitated the implementation of these techniques.^64,65^ Similarly, the widespread availability of LEDs has accelerated their adoption, even though issues related to positioning reproducibility remain. The scientific community is making considerable progress in the use of electrons and photons as green and traceless reagents for the development of enantioselective variants of these processes.

In the context of asymmetric redox transformations, current efforts are focused on replacing conventional co-oxidants and, more critically, the coreductants required to regenerate the oxidation state of the active species within catalytic cycles.^66^ In this respect, established electrochemical and photochemical protocols are increasingly recognized as more sustainable and environmentally benign alternatives, primarily due to their significant reduction in waste generation.

In photochemistry, the use of light-driven asymmetric reactions catalyzed by chiral photocatalysts has attracted considerable interest, though examples remain limited. The main challenges lie in the short lifetimes of excited chiral catalysts and the rapid kinetics of photochemical reactions, which hinder effective stereocontrol. One promising strategy involves the design of chiral photoactive metal complexes with appropriate metal centers and ligands. These complexes have to be photostable, tunable, and versatile, offering enhanced chiral induction across a range of asymmetric transformations. Recent advances highlight their potential as standalone chiral photocatalysts for enantioselective photochemical reactions, eliminating the need for harsh reagents or complex setups. Their intrinsic chiral environments enable efficient stereocontrol in a variety of asymmetric reactions.^67^

The Meggers group has significantly advanced the field by developing chiral-at-metal Ir­(III) or Rh­(III) polypyridyl complexes as efficient standalone photocatalysts.^68,69^ These helically chiral complexes, synthesized using appropriate chiral auxiliaries, are photostable under continuous irradiation and exhibit high enantioselectivity across a broad range of reactions. Their dual functionality-as both enantioselective Lewis acid catalysts and photoredox mediators-combined with synthetic accessibility and resistance to racemization upon photoexcitation, has established them as leading systems in asymmetric photocatalysis, notably in transformations such as the enantioselective alkylation of 2-acylimidazolyl ketones (Scheme 8).^70^

**Scheme 8:**
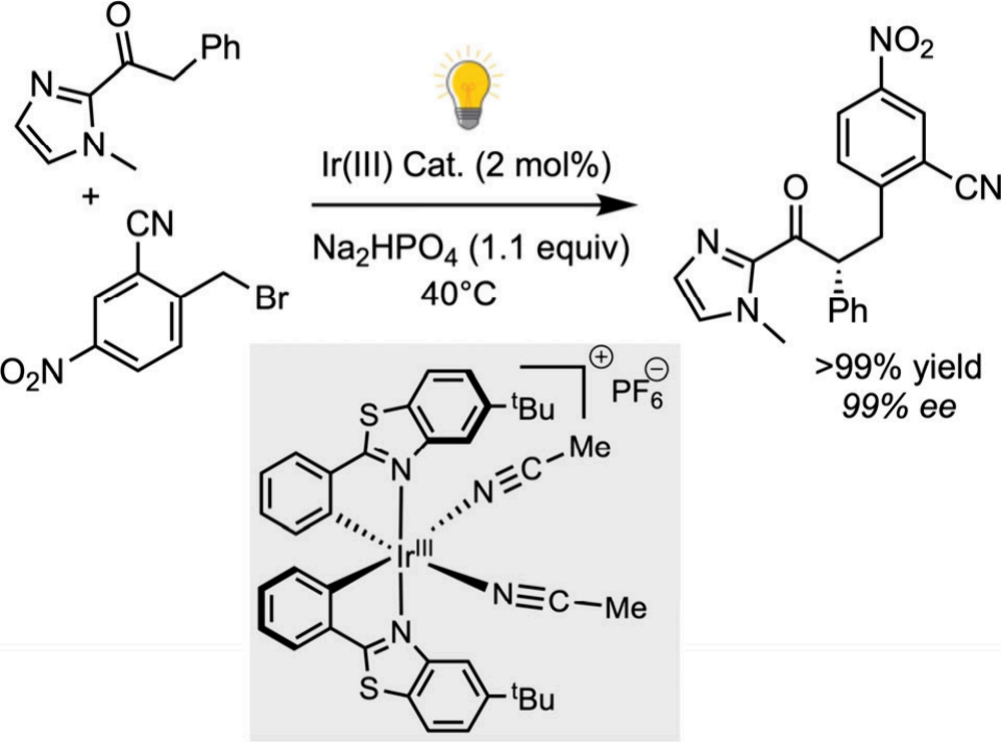
Enantiopure Ir­(III) Chiral-at-Metal-Catalyzed Enantioselective Alkylation of 2-Acylimidazolyl Ketones

A common major drawback about the recent work in that field is the strong reliance on iridium-based photocatalysts to drive the reactions. However, it is conceivable that this negative aspect will soon be mitigated by the recent progress made in the design and development of efficient and robust organic dyes.^71^

In parallel, due to their abundance, low cost, and ability to provide strong photoexcited reducing power copper, nickel and cobalt complexes have naturally been investigated as standalone photocatalysts.^72^ Indeed, developing photocatalysts with similar efficacy that rely on more cost-effective, earth-abundant first-row transition metals remains a major challenge. Most complexes of these metals, particularly nickel, possess significantly different photophysical properties, which limit their ability to function as effective photocatalysts.^73^ As a result, there are only a few reported examples of chiral nickel-based photocatalysts to date.

For these photocatalytic processes involving the generation of radicals, harsh sacrificial reagents or complex setups are typically employed.^74^ However, the excessive consumption of, for example, presynthesized Hantzsch reductants raises significant cost and atom economy concerns. To address the sustainability challenge, the scientific community is shifting toward the use of ubiquitous, “greener” chemical reductants. In this context, very recently a cobalt-catalyzed asymmetric reductive coupling reaction between aromatic aldehydes and aryl iodides has been developed using *i*-Pr_2_NEt as the reducing agent, which is a more accessible and cost-effective product (Scheme 9). This approach affords a wide range of diarylcarbinols isolated with good yields (up to 96% yield) and excellent enantioselectivities (up to 98% ee).^75^

**Scheme 9:**
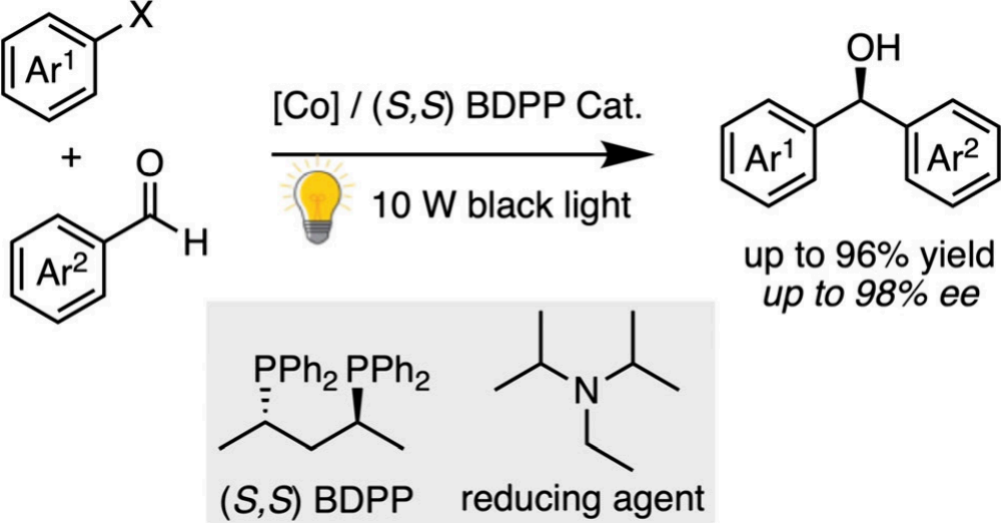
Co-Catalyzed Asymmetric Coupling of Aromatic Aldehydes and Aryl Iodides Using a Simple Amine Reductant

Today, this light-driven strategy represents a more sustainable and complementary avenue for the construction of highly significant chiral molecules.

Despite substantial progress in asymmetric catalysis, major challenges remain, including dependence on stoichiometric oxidants or reductants to regenerate active catalytic species, which often lack environmental compatibility. In this context, organic electrochemistry has emerged as a sustainable and cost-effective alternative.^66^ Compared to conventional methods, electrocatalysis proceeds under milder conditions and typically avoids hazardous oxidants or metal-based reductants. Activation of organic substrates can occur directly via anodic or cathodic processes, or indirectly through redox modulation of metal catalysts. Moreover, the precise control over current and potential allows fine-tuning of electron transfer steps, offering significant advantages in complex multielectron transformations.^76^

The first breakthrough in asymmetric electrochemical catalysis dates back to 1967, when Grimshaw et al. reported the enantioselective reductive synthesis of coumarin using sparteine as a chiral auxiliary and a mercury cathode in a divided cell.^77^ Despite modest yields and enantioselectivities, this work marked the first integration of chiral induction with electrochemistry, laying the foundation for asymmetric electrocatalysis. Since then, rapid advances in the field of transition metal catalysis have sparked growing interest in the synergistic use of electrochemistry and metal catalysts in organic synthesis.

Despite advances in the field of electrosynthesis, the overall development of asymmetric versions remains limited, since its inception.^78^ It is only in recent years, thanks to continuous advances in technology and electrocatalytic equipment, that asymmetric electrochemical catalysis has seen gradual progress and shown promise in a range of applications, including green synthesis and sustainable chemistry.

In the field of asymmetric electrocatalytic oxidation reactions, Amundson and Balko were the first to describe an electrochemically catalyzed Sharpless asymmetric dihydroxylation, using K_3_Fe­(CN)_6_ as a redox mediator to replace traditional chemical oxidants with electrochemical oxidation.^79^ A divided electrochemical cell was used to prevent the cathodic reduction of ferricyanide and osmium metal deposition. However, challenges such as the high toxicity of OsO_4_, limited substrate scope, and insufficient enantioselectivity remained unresolved. In 1996, Torii made significant advances in the Sharpless asymmetric dihydroxylation reaction by replacing K_3_Fe­(CN)_6_ with I_2_ as the redox mediator with potassium osmate salts (Scheme 10).^80^ These optimizations not only reduced toxicity but also expanded the substrate scope and simplified the electrochemical setup, marking a crucial step toward the green, sustainable, and practical development of asymmetric oxidation reactions.

**Scheme 10:**
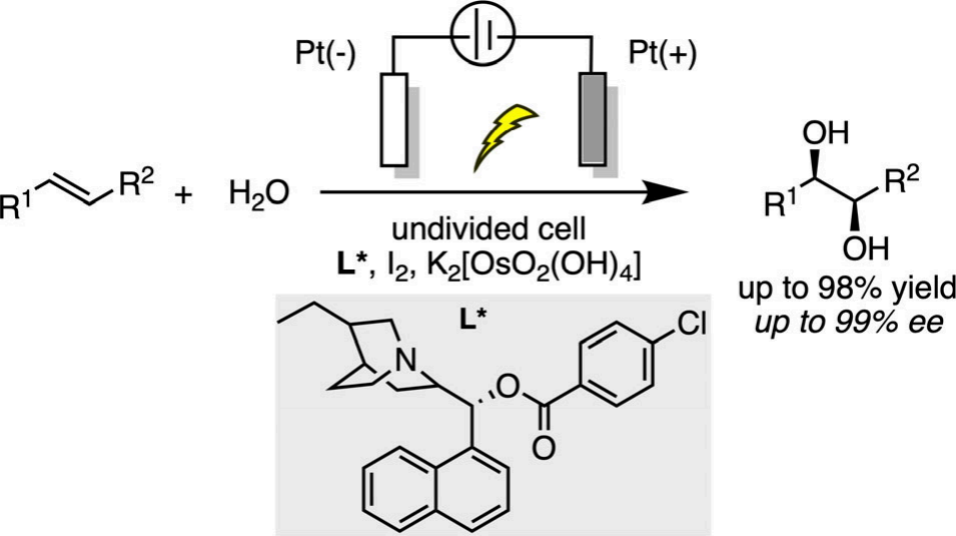
Electrochemically Catalyzed Sharpless Asymmetric Dihydroxylation

Olefin epoxidation using chiral salen complexes has been widely studied, but most methods require stoichiometric oxidants, limiting their green chemistry potential. In 2001, Tanaka’s group developed an electrocatalytic asymmetric epoxidation system in a biphasic setup, combining aqueous sodium chloride and dichloromethane.^81^ Chloride ions are oxidized at the anode to form hypochlorite (ClO^–^), which activates the manganese catalyst to its oxide form. This active species then transfers oxygen to the olefin, producing an asymmetric epoxide. This approach eliminates the need for stoichiometric oxidants, thereby improving the ecocompatibility of the reaction and offering a novel application for electrochemical catalysis in asymmetric synthesis (Scheme 11).

**Scheme 11:**
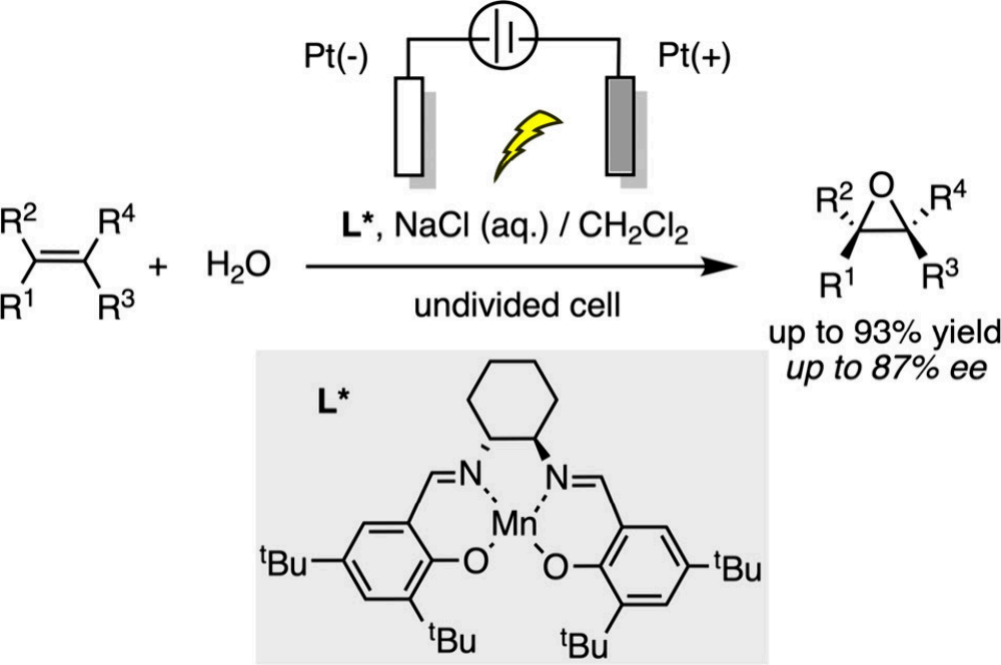
Electrochemical Olefin Epoxidation Using Mn-Chiral Salen Complex

Anodic oxidation reactions have historically progressed slowly, but in recent years, the integration of traditional chemical catalysis with electrochemical asymmetric metal catalysis has gained significant attention, driven by advancements in electrocatalysis and organic synthesis. Electrochemical techniques have enhanced traditional asymmetric metal catalysis, improving reaction efficiency, selectivity, and sustainability. As research in this interdisciplinary field advances, its popularity continues to grow, opening new avenues for the green and efficient development of asymmetric catalytic reactions.

A new family of chiral bisoxazolines (BOX) derived from serine possessing auxiliary coordination sites was developed and applied in the copper catalyzed asymmetric electrochemical difunctionalization of alkenes.^82^ The advantage of this reaction lies in the fact that copper participates in two consecutive electrochemical oxidative cycles, enabling a relay catalytic mechanism. In the first cycle, the copper catalyst generates radicals, while in the second, it facilitates asymmetric cyanation through metal insertion and reductive elimination, ultimately leading to the synthesis of chiral cyano-functionalized products.

In 2022, Shi’s group reported a cobalt-catalyzed enantioselective C–H dehydroalkoxylation and amination enabled by a chiral salicyloxazoline (Salox) ligand.^83^ The method exhibits broad substrate scope and high enantioselectivity. However, its reliance on stoichiometric silver carbonate as the oxidant limits sustainability. To address this, the group developed an electrochemical variant, achieving C–H dehydroalkylation under chemical oxidant-free conditions (Scheme 12).^84^

**Scheme 12:**
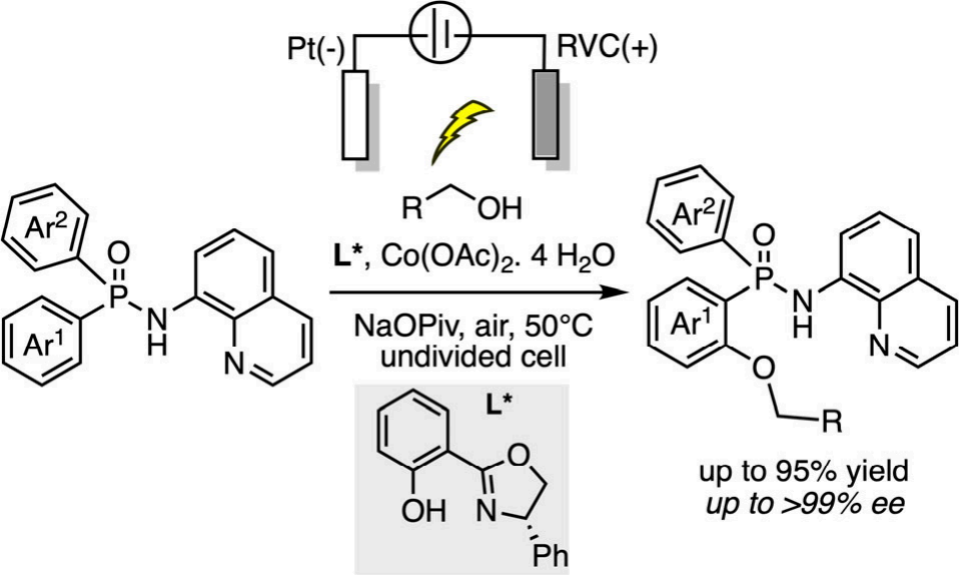
Electrochemical C–H Dehydroalkylation under Chemical Oxidant-Free Conditions

Mechanistic studies based on the work described by the group of Ackermann^85^ led to the conclusion that the cobalt catalyst operates through a III/IV/II redox cycle. It is activated by electrochemical oxidation to generate a Co­(III), which is then subjected to C–H activation and further electrochemical oxidation leads to the target products. At the same time, Ackermann and co-workers demonstrated the versatility of such cobalt-based electrooxidative catalysis, beyond oxygenation reactions.^86^ C–H and N–H annulations from carboxylic amides successfully yielded enantioenriched spirolactams, dihydroisoquinolinones, and axially chiral compounds. This dehydrogenative activation process allowed also for the formation of various phosphorus-stereogenic molecules, by desymmetrization, using simple ligands derived from BINOL or amino alcohols.

In the field of asymmetric electrocatalytic reduction reaction, Reisman’s group reported a nickel-catalyzed enantioselective reductive cross-coupling of alkenyl and benzyl halides using manganese as a stoichiometric reductant and a chiral BOX ligand.^87^ Despite high enantioselectivities and a broad substrate scope, the method suffered from poor reproducibility due to inconsistent metal reactivity and stirring issues, alongside substantial metal waste. To overcome these limitations, an electrochemical variant was developed in 2019, replacing Mn(0) with a reticulated vitreous carbon (RVC) cathode and a sacrificial zinc anode.^88^ The addition of NaI as a supporting electrolyte both improved conductivity and activated electrophiles via halide exchange, significantly enhancing reaction efficiency and stereocontrol (Scheme 13).

**Scheme 13:**
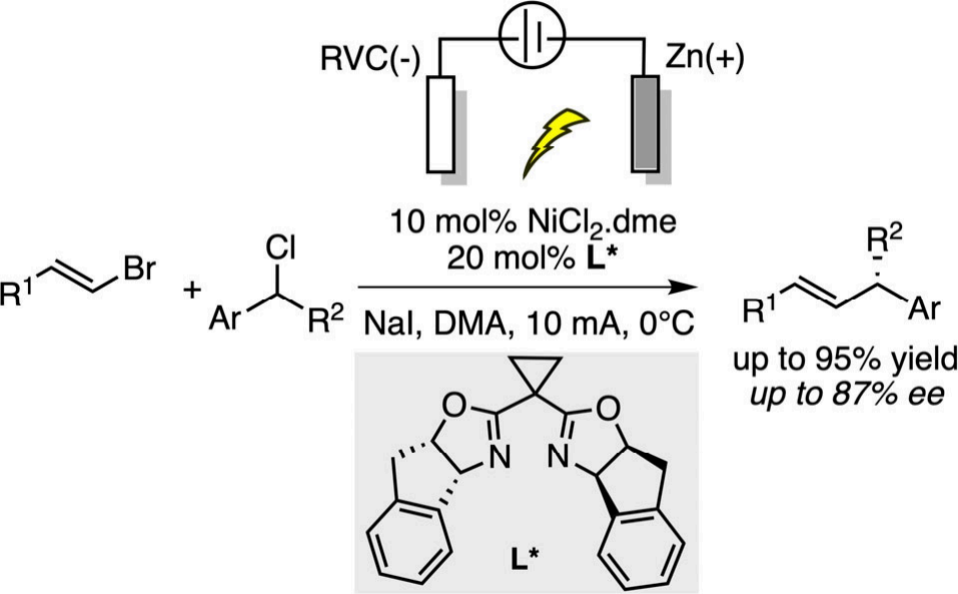
Nickel-Electrocatalyzed Enantioselective Reductive Cross-Coupling of Alkenyl and Benzyl Halides

In 2020, Mei’s group developed an efficient enantioselective electrochemical reductive coupling for the synthesis of BINOL derivatives, achieving high yields and enantioselectivities.^89^ In contrast, the same transformation using only manganese as a reductant, without electrochemical input, resulted in significantly lower yields. This highlights the enhanced reactivity, selectivity, and controllability of the electrochemical process. The method also offers high atom economy and aligns well with principles of sustainability.

These electrocatalytic nickel-catalyzed asymmetric cross-couplings allowed avoiding the use of external reducing agent; nevertheless; they all required the presence of a sacrificial anode, limiting their sustainability. To overcome these drawbacks, the Nevado group developed a nickel-catalyzed asymmetric electrochemical cross-coupling between aryl aziridines and alkenyl bromides, using triethylamine as a terminal reductant eliminating the need for a sacrificial anode (Scheme 14).^90^ This approach offers excellent enantioselectivity (up to 96:4 er), broad substrate scope, and high functional group tolerance under mild conditions. Mechanistic studies revealed that the reaction proceeds via nucleophilic halogenation-induced ring opening of aziridines to form radical intermediates, followed by nickel-catalyzed electron transfer and reductive elimination. This work introduces a sustainable and efficient strategy for the synthesis of chiral amines and sets the stage for further developments in asymmetric electrochemical reductive couplings.

**Scheme 14:**
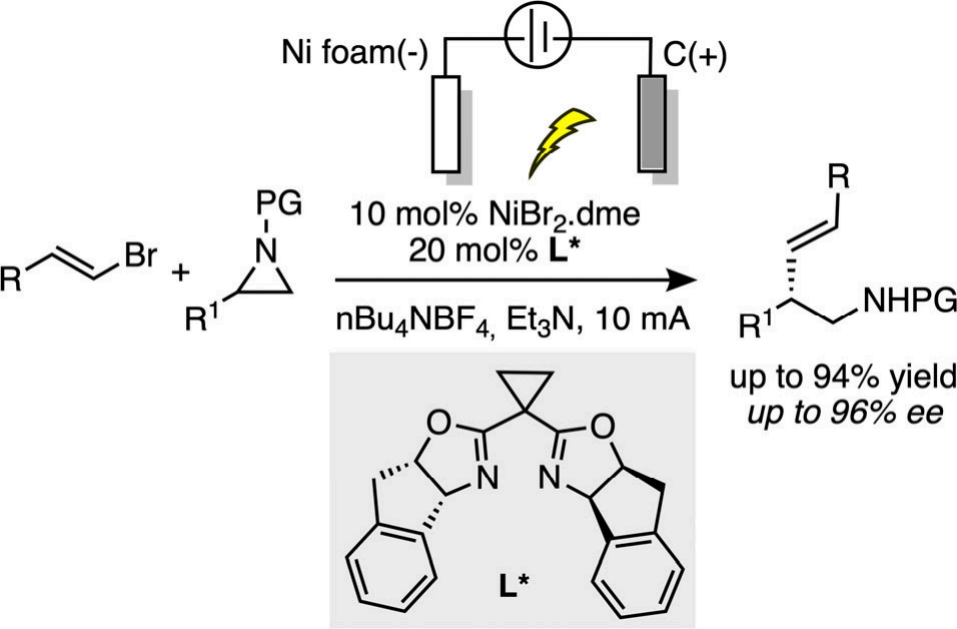
Use of Et_3_N as an Organic Sacrificial Reagent Preventing Metallic Waste from a Sacrificial Anode

The Lu group recently introduced a paired electrolysis strategy combining anodic C­(sp^3^)–H activation with cathodic reduction and chiral Lewis acid catalysis for the enantioselective alkylation of sulfonimides.^91^ This method enables efficient cross-coupling between benzyl and sulfonimide radicals, achieving high yields (up to 88%) and excellent enantioselectivities (up to 96% ee) across diverse substrates. Mechanistic studies confirmed that anodic oxidation selectively generates benzyl radicals, while cathodic reduction forms sulfonimide radicals. These intermediates undergo asymmetric coupling under chiral Lewis acid cocatalysis.

Progress toward more sustainable methods for preparing chiral compounds naturally also involves the use of new technologies. In particular, flow processes have been effectively transferred from bulk to fine chemistry.^92^ Numerous examples have been reported in the literature, clearly demonstrating the usefulness of a compact bed reactor containing immobilized chiral catalysts for optimizing separation, combined with advantageous automation, easy or no purification, and possible long-term and/or varied use of the catalyst batch. For example, mass-transfer between heterogeneous catalysts and H_2_ has been improved in the enantioselective hydrogenative enyne cyclization promoted by the use of supported chiral Rh-catalysts. These complexes were immobilized by ionic interactions on silica composites functionalized by heteropolyacids and amines. Optimization of continuous flow conditions allowed achieving high values of TOF, TON and enantioselectivity, without any leaching of metal traces.^93^ Organocatalysts have also been immobilized and packed in reactors to achieve the continuous formation of valuable enantioenriched compounds demonstrating very high stability. This was recently illustrated by the use of chiral isothiourea supported on Merrifield resin for the enantioselective synthesis of a range of heterocyclic products from Michael addition-cyclization reactions.^94^ This study describes the optimization of reaction conditions, the application of the methodology to a wide range of substrates, and large scale testing, all carried out with the same batch of catalyst. In this field too, innovations in microreactor technology have enabled new developments thanks to the precise control of reaction conditions in these devices. Xu et al. recently tackled the challenge of electrocatalytic asymmetric oxidative transformations, for which the conditions in batch reactors are not optimal in terms of the quantities of electrolytes and other additives required.^95^ Running the reaction in microfluidic electrochemical reactors at a constant current enabled the production of various enantioenriched products through various oxidation strategies (one of which is shown in Scheme 15). This facilitates the scale-up of the process while overcoming the accumulation of sensitive intermediates and all the aforementioned limitations.

**Scheme 15:**
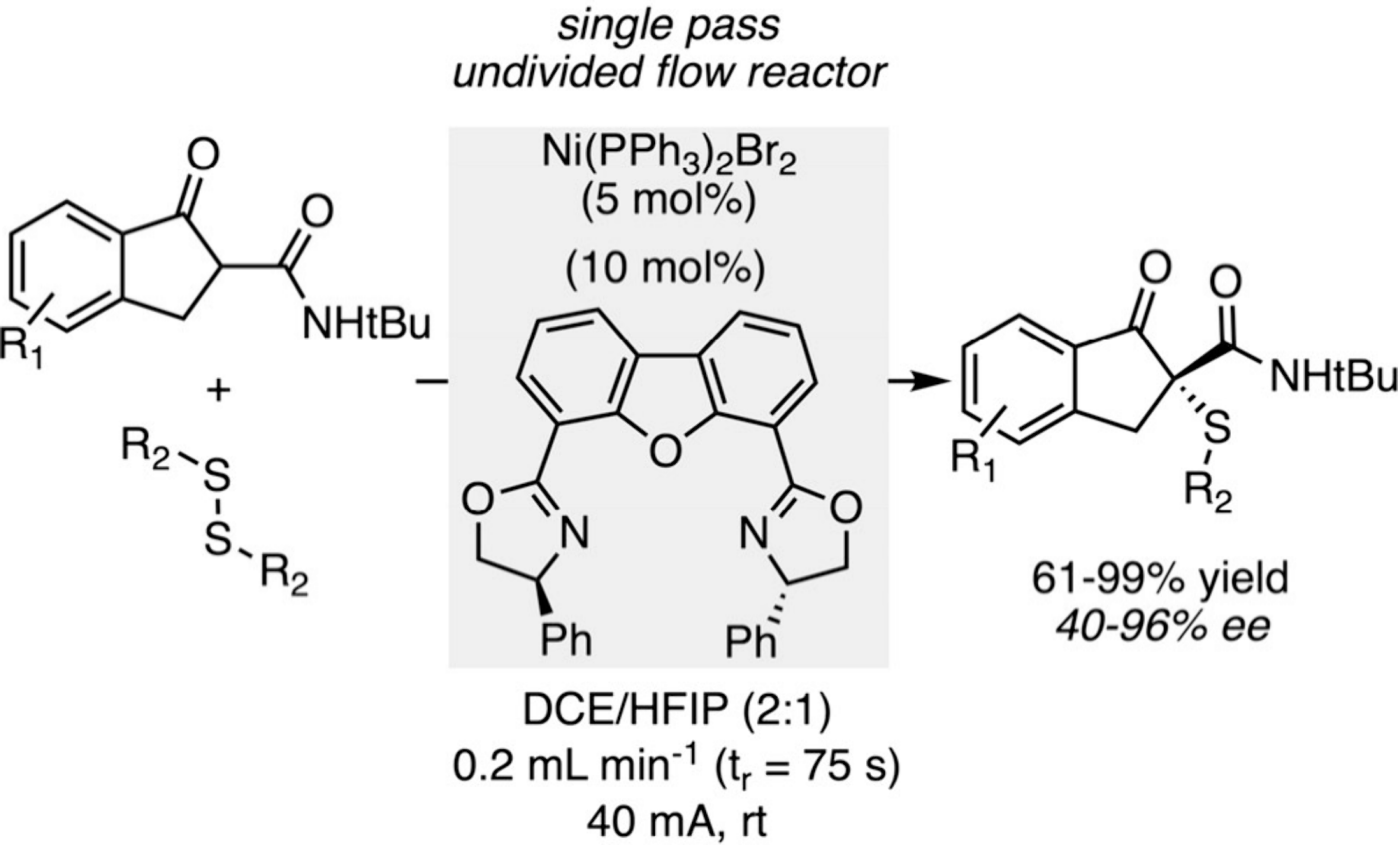
Electrochemical Asymmetric C–H Sulfenylation in Continuous Flow

The shift toward sustainability also involves the use of mechanochemistry, carried out with minimal or without solvent, and asymmetric catalysis is no exception to this rule. The first trials appeared very quickly during the development of asymmetric organocatalysis and demonstrated the value of this method for achieving faster reactions with better-adjusted reagent stoichiometries.^96^ Numerous transformations have since been explored, whether based on covalent interactions or noncovalent activation strategies, leading to enantiomerically enriched target products, generally more rapidly than in solution.^97^ Asymmetric transformations catalyzed by metals in ball mills have also been reported successfully, provided that the reaction conditions are optimized to ensure, for example, the proper initial formation of the chiral complex, which is slower than in solution. The presence and optimization of new reaction parameters, such as grinding frequency, also influence selectivities. As an example, Bolm and his group performed a Michael-type Friedel–Crafts alkylation of indoles, catalyzed by a Cu­(I)-bis­(oxazoline) complex in a mixer bill, which yielded the target products in high yield and good enantioselectivity values, after fine-tuning the reaction conditions (Scheme 16).^98^

**Scheme 16:**
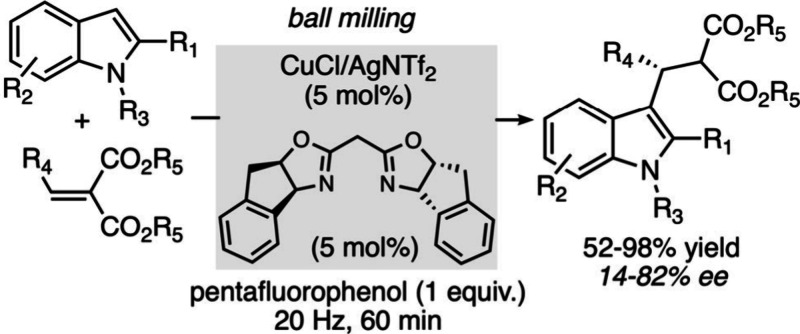
Cu-Catalyzed Asymmetric Friedel–Crafts Alkylations of Indoles in a Ball Mill

The ee values obtained are not always as high as the maximum values obtained in solution when these require low temperatures, but alternating between ball-milling and cooling breaks makes it possible to compensate for these differences to a certain extent. Developments, particularly using twin-screw extrusion^99^ or resonant acoustic mixing^100^ currently not developed for asymmetric procedures, as far as we know, are also expected in this context.

Asymmetric photocatalysis and electrocatalysis are rapidly evolving toward more sustainable paradigms, but several challenges remain. A key priority is the development of catalytic species based on earth-abundant metals or organic chromophores in order to reduce dependence on precious and rare elements. Achieving precise stereocontrol in ultrafast photochemical and electrochemical processes is also a major challenge, requiring new chiral environments capable of guiding high-energy reactive species. Future progress will rely on reducing the use of sacrificial oxidants and reductants, and advancing electrocatalytic methods through robust and reproducible designs. Advances in flow systems, microfluidics, and mechanochemistry are expected to improve efficiency, scalability, and catalyst lifespan, though challenges remain in mass transfer, stability, and reactor standardization. Continued innovation at the interface of catalysis, renewable energy, and reaction engineering is key to making asymmetric catalysis a cornerstone of greener synthesis.

## 4 MULTICATALYSIS

Multicatalytic processes, i.e., processes involving two or more catalysts working in concert in the same pot, can be divided into two categories. Relay catalysis refers to multicatalytic processes where catalytic cycles do not share any catalytic intermediates, i.e., each individual step is promoted by no more than a single catalyst.^101^ On the other hand, cooperative catalysis, also called synergistic catalysis,^102^ refers to multicatalytic systems in which catalysts operate in synergy by sharing catalytic intermediates,^101^ thus enabling the activation of both partners of a bimolecular reaction. Multicatalytic processes are perfectly in line with sustainability issues for obvious reasons as they benefit from every positive aspect of a catalytic transformation but they also have the potential to lead to increased molecular complexity in a one pot fashion.^101^ In that sense, a multicatalytic approach can significantly reduce time, waste and cost of synthetic process.^102^ When one chiral catalyst is engaged in a multicatalytic system, the transformation can be enantioselective and when two or more chiral catalysts are present, it can even be stereodivergent depending on the combination that is selected.^103^

A multicatalytic approach can significantly reduce time, waste and cost of synthetic processes

Relay catalysis offers the opportunity to design multicatalytic cascades with one or more chiral catalysts for a rapid and stereocontrolled access to complex organic scaffolds. This approach can dramatically improve chemical synthesis and has the potential to transform the manufacturing of potent molecules in industry.

A team from Merck process and research development recently reported a striking example to support this statement. Indeed, they designed a biocatalytic cascade involving five engineered enzymes (including two immobilized ones) and four auxiliary enzymes to stereoselectively assemble Islatravir, a potent molecule for HIV treatment, from simple achiral building blocks in 51% overall yield.^104^ In terms of sustainability, this stereoselective sequence proceeds with high step- and atom-economy and takes place under mild conditions in a single aqueous solution without the isolation of intermediates (Scheme 17).

**Scheme 17:**
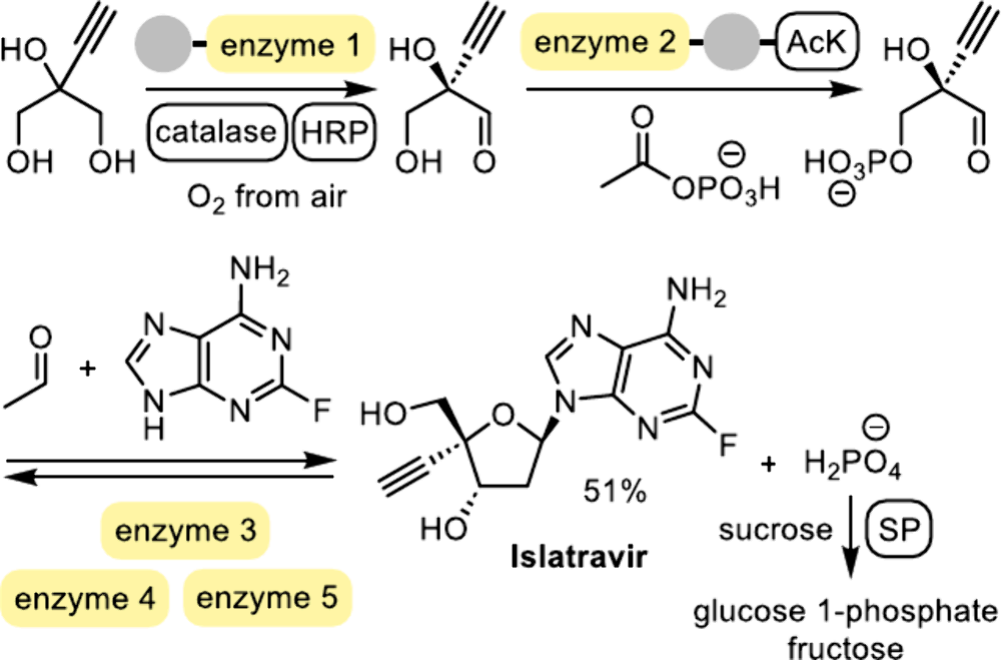
Biocatalytic Cascade for the Synthesis of Islatravir from a Simple Achiral Precursor

Borrowing hydrogen (BH) catalysis is a type of relay catalysis that can mimic some biocatalytic reactions to enable the transformation of cheap and readily available alcohols into high-value molecules via the reversible formation of a reactive carbonyl intermediate mediated by a metal catalyst.^105^ Because BH catalysis typically avoids wasteful and unnecessary stoichiometric redox-state manipulations, it usually leads to eco-compatible processes with high atom-economy and low chemical waste. Multicatalytic systems incorporating a BH catalyst offer the opportunity to engage the carbonyl intermediate into stereoselective transformations to afford valuable enantioenriched molecules directly from commodity alcohols. In that respect, Quintard and Rodriguez combined BH catalysis with organocatalysis to transform allylic alcohols into complex building blocks featuring two contiguous stereocenters, one of which being quaternary.^106^ To promote the BH catalytic cycle, they selected the Knölker complex,^107^ a simple achiral iron catalyst that converts allylic alcohols into sensitive unsaturated aldehydes. These aldehydes are now prone to iminium activation with the Hayashi catalyst,^108^ a common proline-derived organocatalyst, to trigger the stereoselective addition of a β-ketoester. Then, final hydrogenation of the obtained aldehyde affords the desired product and closes the BH catalytic cycle (Scheme 18).

**Scheme 18:**
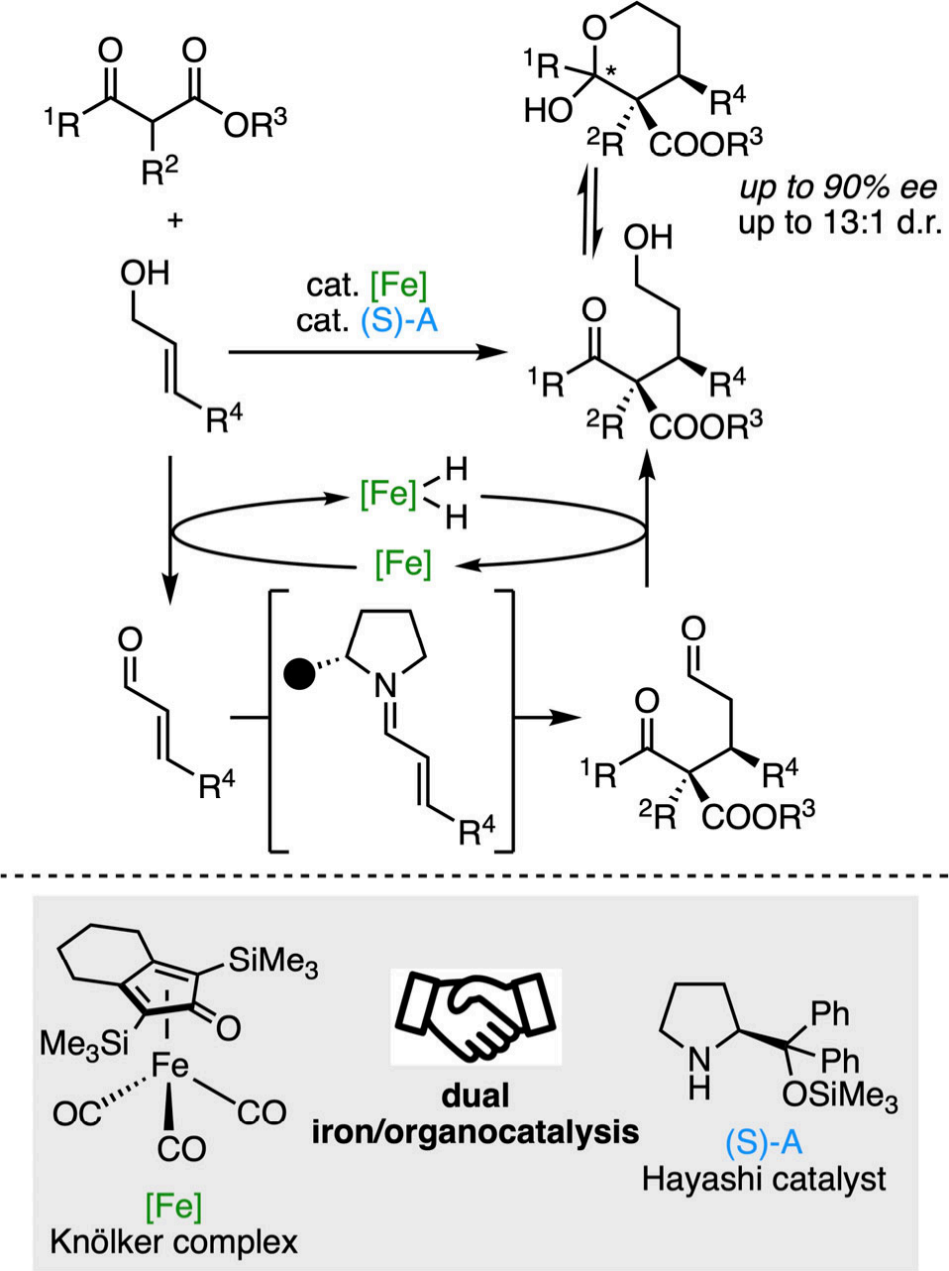
Stereoselective Functionalization of Allylic Alcohols by BH Catalysis

In a similar fashion, Wang and co-workers recently reported a dual catalytic process involving a chiral borrowing hydrogen catalyst together with a chiral copper catalyst acting as a Lewis acid.^109^ The combination of these two chiral catalysts enabled the stereodivergent hydroalkylation of racemic allylic alcohols with prochiral nucleophiles leading to valuable nitrogen-containing heterocycles having two distal stereocenters. Each of the four stereoisomers is accessible with this method, it just depends on the combination of the catalysts that is used.

Cooperative catalysis, also called synergistic catalysis,^110^ refers to multicatalytic systems in which catalysts operate in concert by sharing catalytic intermediates, thus enabling the activation of both partners of a bimolecular reaction. Cooperative catalysis is a powerful strategy to develop novel and previously unattainable chemical transformations because of the simultaneous dual activation of two reactions partners, a feature that can significantly decrease the activation barrier of a given transformation. Along this line, cooperative catalysis allows for example the enantioselective hydrogenation of imines to amines, an industrially relevant process, with an environmentally benign iron catalyst working in concert with a chiral phosphoric acid.^111^ Such type of hydrogenation is traditionally performed with monocatalytic systems involving expensive and nonsustainable chiral complexes based on a precious transition metal such as Ir, Rh or Ru.^112^ In comparison, complexes based on earth abundant iron are less performant for hydrogenation^113^ and much fewer chiral ligand scaffolds are available to perform it in an enantioselective fashion. However, since cooperative catalysis enables the activation of both partners, it opens the doors for novel strategies. In this respect, Beller and co-workers relied on the Knölker complex^111^ to activate dihydrogen while a chiral phosphoric acid is used to activate the imine partner toward the iron-hydride, thus enabling enantioselective hydrogenation without a chiral ligand on the metal (Scheme 19).

**Scheme 19:**
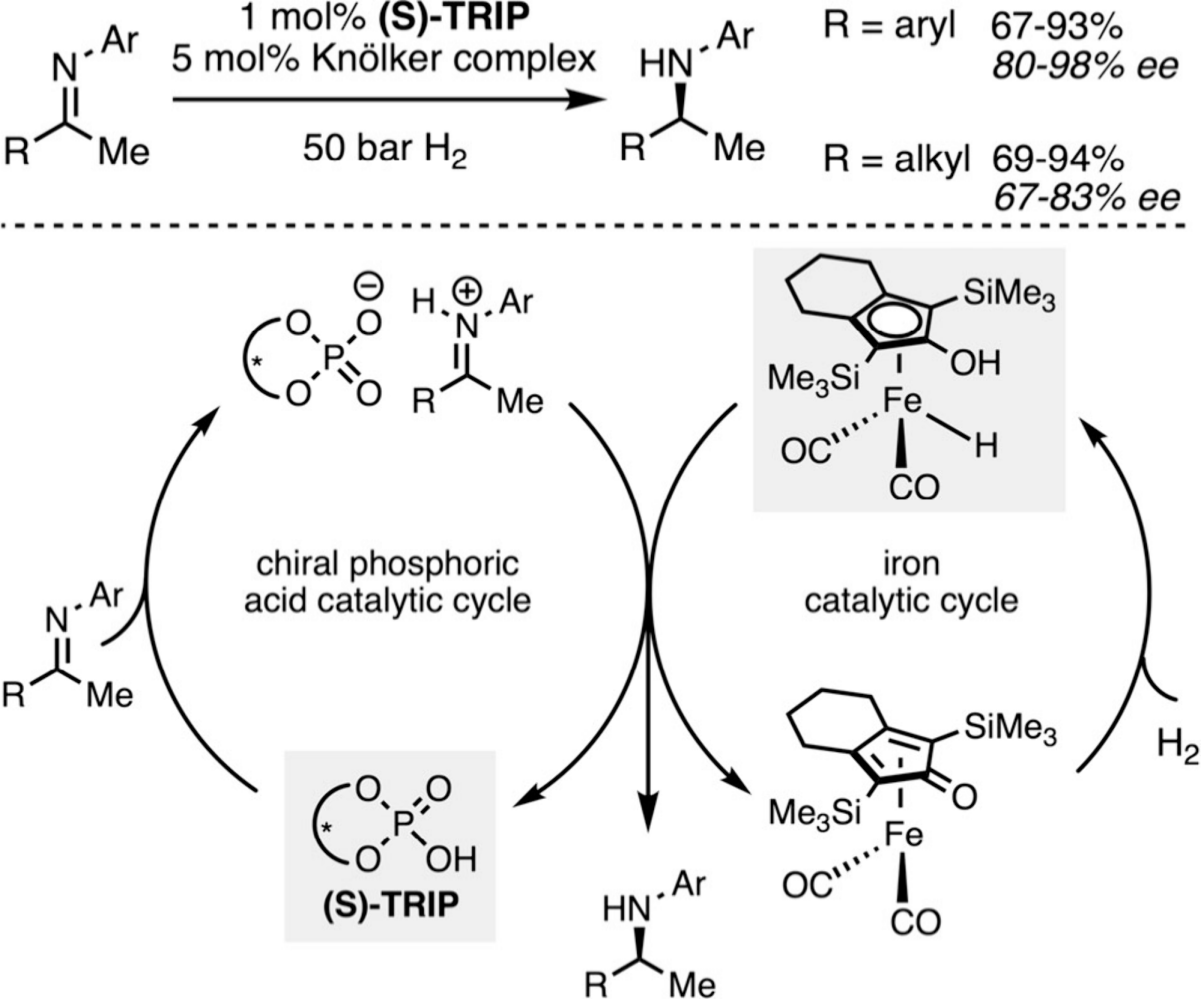
Enantioselective Hydrogenation of Imines with a Cooperative Iron/Chiral Phosphoric Acid Catalytic System

Another type of cooperative catalysis that looks very promising for sustainable synthesis is the merger of photocatalysis and enantioselective catalysis.^114^ In these multicatalytic systems, a photocatalyst acts like an antenna to harvest light energy and convert it into chemical energy via the generation of short-lived open-shell species. Meanwhile, a chiral catalyst orchestrates stereoselective processes with these species to afford enantioenriched molecules. Recent work from the group of Phipps on enantioselective Minisci reactions with redox active esters illustrates the great potential of such dual enantioselective catalytic systems (Scheme 20).^115,116^ Indeed, they managed to transform a variety of pyridines, quinolines and diazines, which are among the most important nitrogen containing heterocycles for synthetic bioactive compounds,^117^ into highly valuable benzylic amine motifs under very mild conditions (room temperature, neutral conditions). An excited iridium-based photocatalyst (Ir-**PC**) reduces activated esters derived from amino acids to generate α-amino radicals via a radical decarboxylation. In the meantime, a chiral phosphoric acid (**CPA**) protonates a nitrogen-containing heterocycle to promote a regioselective addition of the radical followed by an enantiodetermining deprotonation. The reaction usually proceeds with excellent regio- and enantioselectivities, thus enabling selective late-stage peripheral editing of potent pharmaceuticals.^115^ Of note, other research groups could later advantageously replace the iridium photocatalyst used in the original system with an organic dye^118^ or a combination of triphenylphosphine and iodine.^119^

**Scheme 20:**
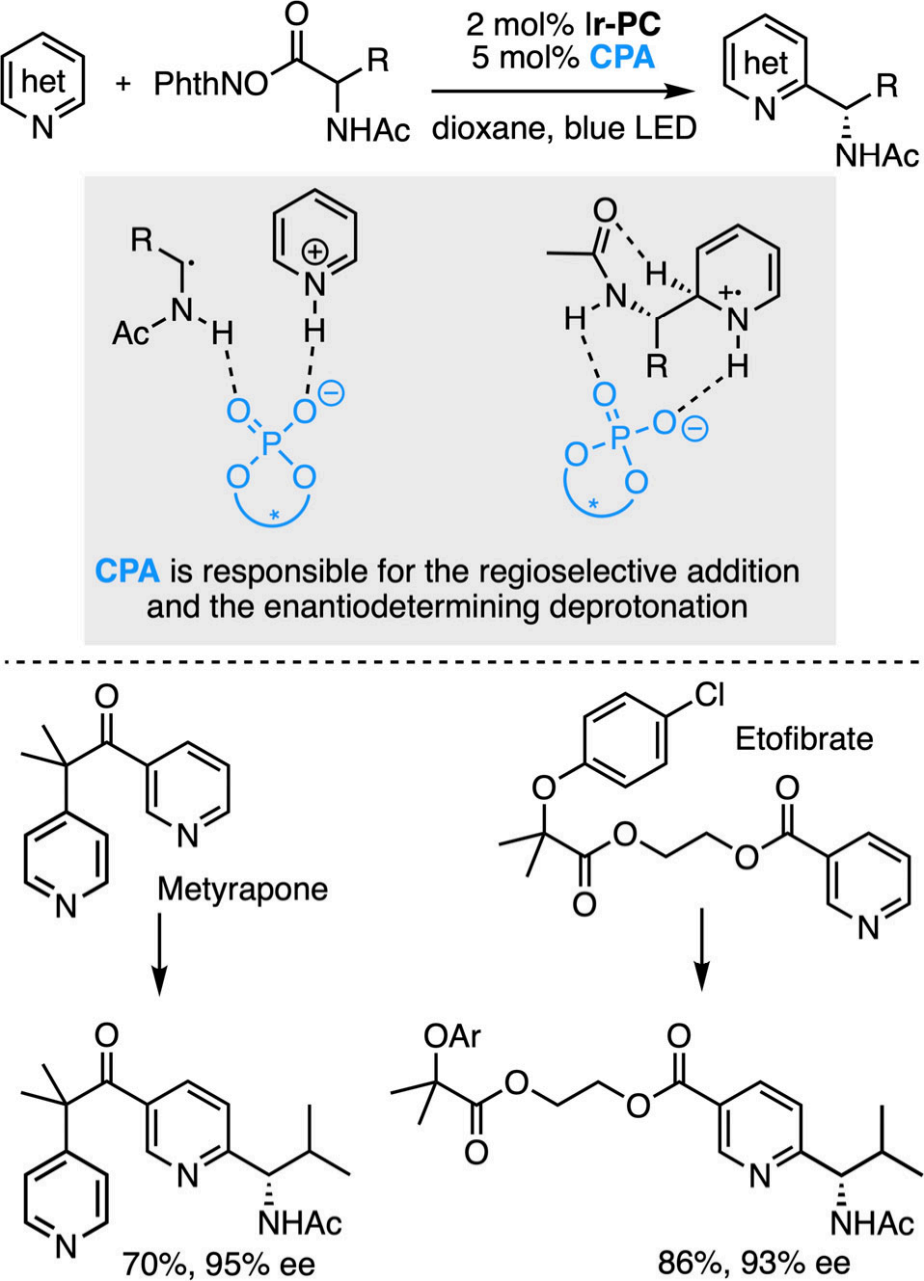
Enantioselective Minisci Reaction with a Dual Photocatalytic System

Because light energy is injected in dual photocatalytic systems, activation barriers can be drastically reduced so it opens tremendous opportunities for sustainable enantioselective catalysis. For example, one could consider replacing a noble metal such as palladium by a more sustainable but less performant one such as copper^120^ or nickel^121^ for novel enantioselective cross couplings and such type of processes can even be run in flow.^122^ Because the generation of radicals in a controlled manner is greatly facilitated by photoredox catalysis, one can think about novel enantioselective C–H functionalizations operating *via* hydrogen atom transfer (HAT) pathways to access molecular complexity from renewable ressources.^123^ Finally, photocatalysis also allows for contra-thermodynamics processes such as deracemization^124^ or forbidden cycloadditions,^125^ transformations that can result in high-value enantioenriched compounds starting from simple and readily available substrates.

## 5 STEP-ECONOMY AND MOLECULAR DIVERSITY

Recent trends in chemistry require organic chemists to design new strategies for a more efficient organic synthesis. A primary objective is to develop approaches that enable a rapid access to increasingly complex molecules. Today, total synthesis is envisaged with the aim of preparing natural products on a gram scale in a minimum number of steps, so that the quantities supplied facilitate the study of their properties.^126^ On the other hand, the direct site-selective modification of a complex molecule, such as a natural product or a drug, is an invaluable method to provide a streamlined access to libraries of compounds, that will be too time-consuming or even impossible to prepare using a classical organic chemistry. Such transformations can be likened to a scalpel aimed at modifying the properties of molecules in a customized manner and reaching a new molecular space. They include the peripheral editions of a compound through late-stage functionalization,^127^ or its skeletal modification via rearrangements or atom insertion, deletion or swapping.^128^

Natural products still challenge the creativity of organic chemists because the field of total synthesis has evolved to be included in translational research programs. In this context, the main objective is now to produce a natural product on a scale that allows its properties to be studied. This requires the design of streamlined synthetic schemes, and catalytic asymmetric synthesis appears essential to this end. The total synthesis of (+)-Lucidumone recently published by the group of de la Torre perfectly highlights such trends in modern asymmetric total synthesis (Scheme 21).^129^ The retrosynthetic analysis led the authors to propose an inverse-electron-demand Diels–Alder cycloaddition between a 2-pyrone and an enol ether as the key step of the sequence. Thus, the search for an enantioselective synthesis of (+)-Lucidumone required developing this step in an asymmetric version, which was made possible by the discovery of a versatile copper-catalyzed process.^130^ The catalytic enantioselective cycloaddition delivers the expected bridged lactone with high levels of diastereo- and enantiocontrol. Then, the lactone was converted in 9 steps to the enantiopure natural product with an overall yield of 27% that enables the isolation of the enantiopure natural product on a gram quantity.

**Scheme 21:**
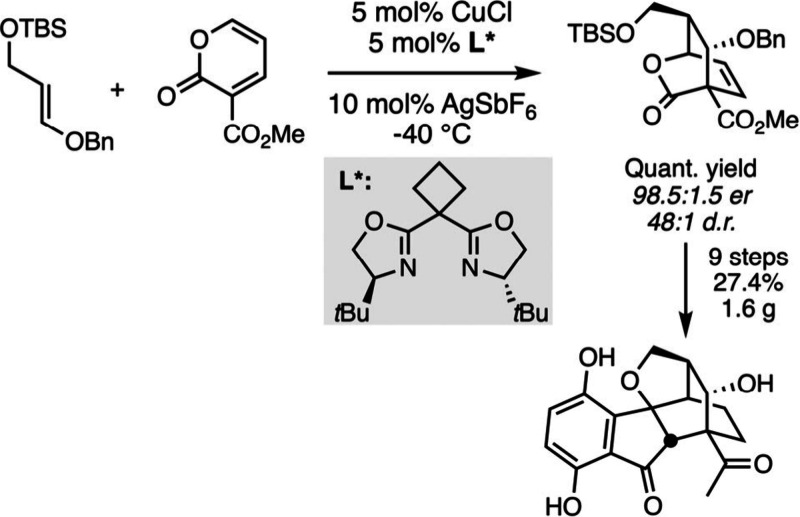
Catalytic Asymmetric Total Synthesis of (+)-Lucidumone

The pressing need for synthetic efficiency including step- and atom-economy has translated to the emergence of new challenges in asymmetric catalysis.

A typical example is represented by catalytic C–H functionalization reactions, which today lead the organic chemists to consider nonactivated C–H bonds as functional groups.^131^ This paradigm shift affords new retrosynthetic analyses for a more efficient synthesis, and for which the design of asymmetric versions requires the development of new chiral catalysts. Needless to mention, the engineering of new bioinspired catalysts provides unique opportunities to this end, given the ability of natural enzymes to catalyze highly selective C–H oxidation reactions.^132^

A first class of transition metal-catalyzed C–H functionalization reactions proceeds via an inner sphere mechanism. These so-called “C–H activation” reactions have challenged the creativity of chemists who have designed new families of ligands to control the formation of stereogenic centers.^133^ This approach was recently applied to the control of the more challenging helicoidal chirality for the enantioselective synthesis of carbo­[*n*]­helicenes and the development of OLEDs (Scheme 22).^134^ Thus, the group of Baudoin described a catalytic system based on a Pd(0) complex and a binaphthyl-derived phosphine-carboxylate ligand L*. This bifunctional ligand plays a key role in each elementary step of the asymmetric transformation, which includes the oxidative addition of the Pd(0) complex into the C–Br bond, the subsequent concerted-metalated deprotonation of the C–H bond by the carboxylate moiety, and the final reductive elimination to deliver the helicene. Particularly, this ligand proved more efficient than a simple binaphthylphosphine combined with an external carboxylate cocatalyst, to convert achiral (*Z*)-1,2-diarylethylenes to enantioenriched helicenes via a palladium-catalyzed intramolecular C–H arylation reaction. The complexity of the phosphine-carboxylate ligands raises the issue of their multistep synthesis, however, these are noteworthy ligands as they proved as efficient for the equally challenging control of planar chirality in the formation of cyclophanes through an intermolecular C–H arylation reaction.^135^

**Scheme 22:**
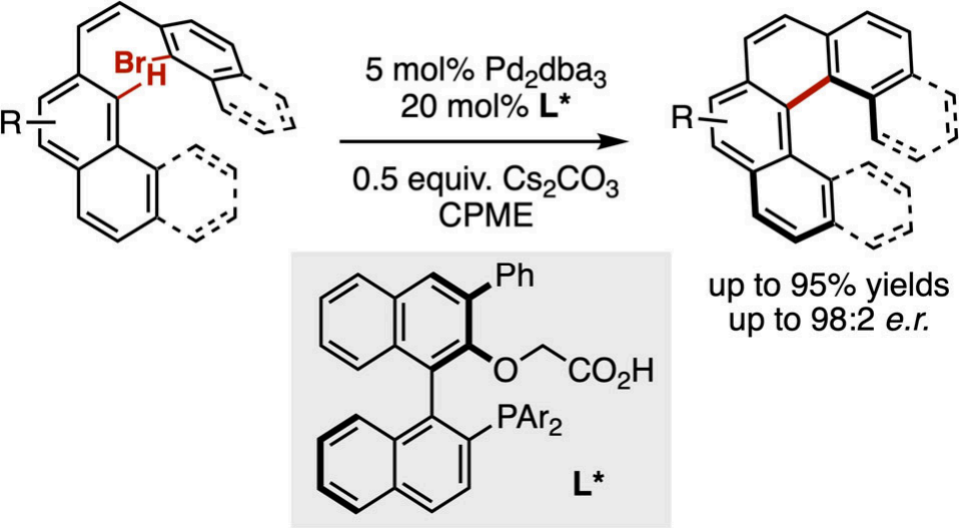
Palladium-Catalyzed Enantioselective Synthesis of Carbo­[*n*]­helicenes

Metal-catalyzed C–H functionalization reactions can also proceed according to an outer sphere mechanism with the formation of highly reactive metal-bound oxo, carbene or nitrene species that directly insert into a C­(sp^3^)–H bond. These transformations, which are reminiscent to those catalyzed by cytochromes in Nature, have a complementary scope to that of C–H activation processes.^136^ It is noteworthy that, contrary to the latter, they do not rely on the use of a directing group and their selectivity can be controlled through the design of relevant catalysts. The use of dirhodium tetracarboxylates, to this end, proved remarkably efficient.^137^ According to the carboxylic acid ligand, they can adopt various geometries. Among those, the *C*_4_-symmetrical complexes offer unique opportunities in asymmetric catalysis with their chiral hydrophobic pocket delineated by the ligands that enables the establishment of noncovalent interactions between the substrate and the reagent. The group of Davies demonstrated that this chemistry was perfectly suited to access a new molecular space in medicinal chemistry (Scheme 23). Thus, the catalytic enantioselective insertion of rhodium-bound carbenes generated from diazo compounds into the tertiary C­(sp^3^)–H bond of strained bicyclo[1.1.1]­pentanes affords a direct access to optically pure derivatives.^138^ This approach competes favorably with previous synthetic strategies in terms of step-economy, affording in one step bioisosteric analogs of phenylglycines with improved pharmacokinetic properties.

**Scheme 23:**
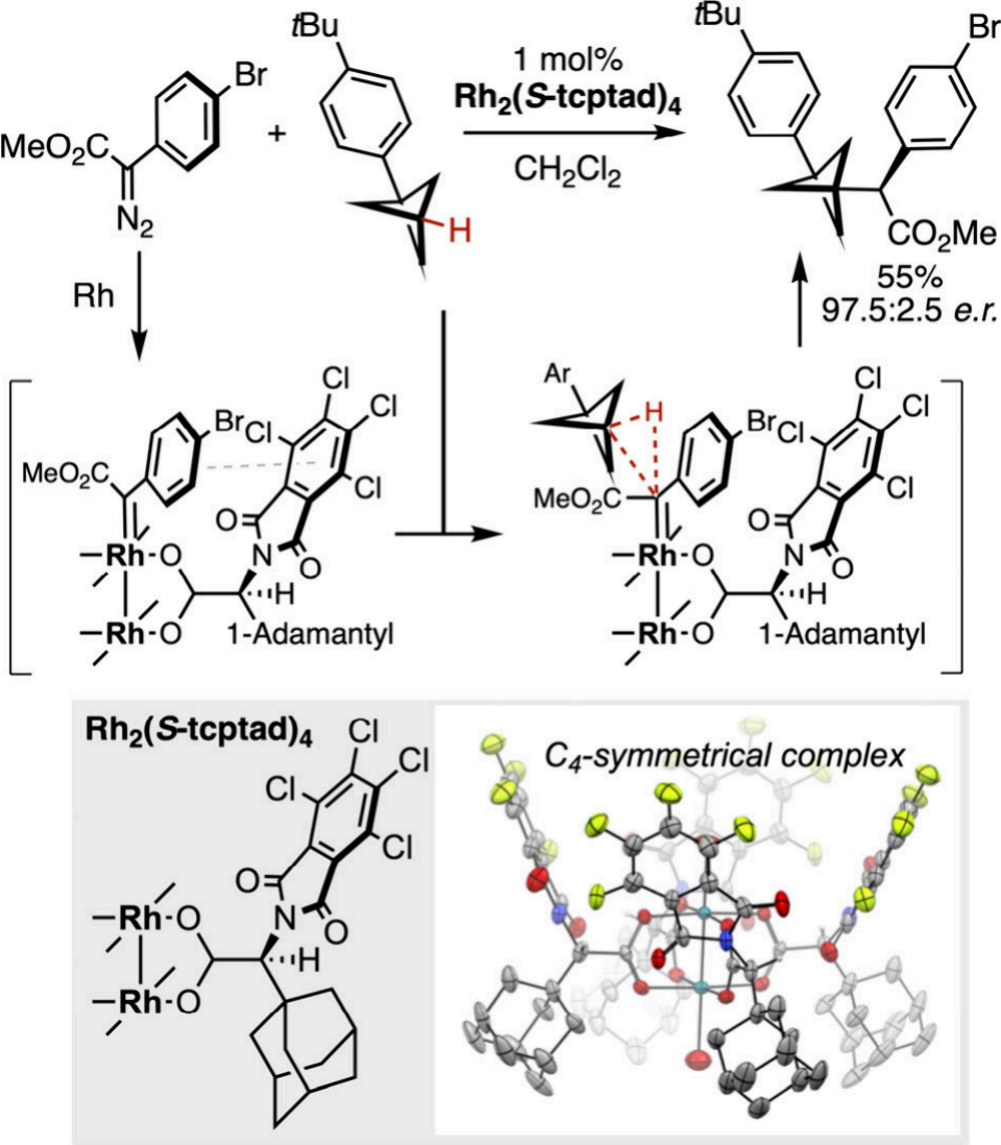
Rhodium­(II)-Catalyzed Asymmetric Functionalization of Bicyclo[1.1.1]­pentanes

In the context of catalytic nitrene C–H insertion reactions, the *C*_4_-symmetrical hydrophobic pocket of the dirhodium complexes is appropriate to tune the selectivity of the reaction according to the nature of the reagent (Scheme 24). Whereas the BDE-driven enantioselective amination of benzylic C–H bonds (BDE 85 kcal·mol^–1^) can be performed efficiently with a benzylic sulfamate,^139^ the use of a trisubstituted aromatic sulfamate enables the asymmetric conversion of an unactivated homobenzylic C–H bond (BDE > 95 kcal·mol^–1^).^140^ Such a catalyst-controlled strategy is invaluable to create a divergent molecular diversity from a single molecule.

**Scheme 24:**
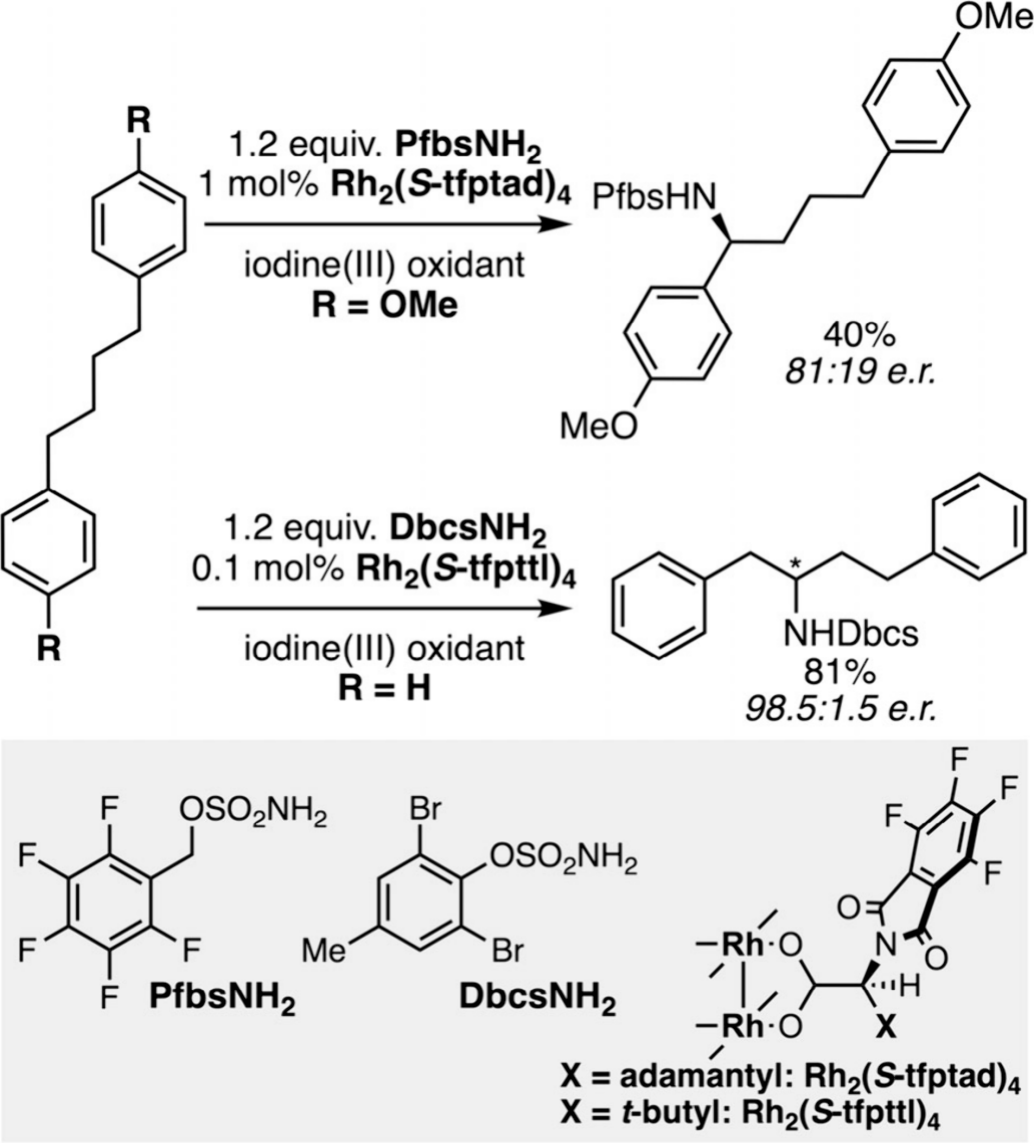
Catalyst-Controlled Selective C–H Amination Reactions

Highly selective C–H functionalization reactions can finally be designed by application of radical chemistry, a field that recently emerged with the advent of photocatalysis (see previous chapter).^141^ Combined with transition metal catalysis, this strategy provides new opportunities in asymmetric synthesis for the late-stage functionalization of bioactive compounds. This was demonstrated by the group of Kramer who reported the use of a chiral copper complex combined with an iridium photocatalyst as an efficient system for the enantioselective amination of benzylic C­(sp^3^)–H bonds (Scheme 25).^142^ The reaction proceeds through the formation of a benzylic radical via a hydrogen atom transfer mediated by the *tert*-butoxy radical generated from di-*tert*-butylperoxide in the presence of the photocatalyst. The radical then reacts with a chiral copper-amido species to deliver the expected benzylic amine. Various drug-like molecules can be selectively functionalized under these conditions, a transformation whose value is greatly enhanced by the possibility of using ^15^N-labeled reagents for the synthesis of radiolabeled compounds.

**Scheme 25:**
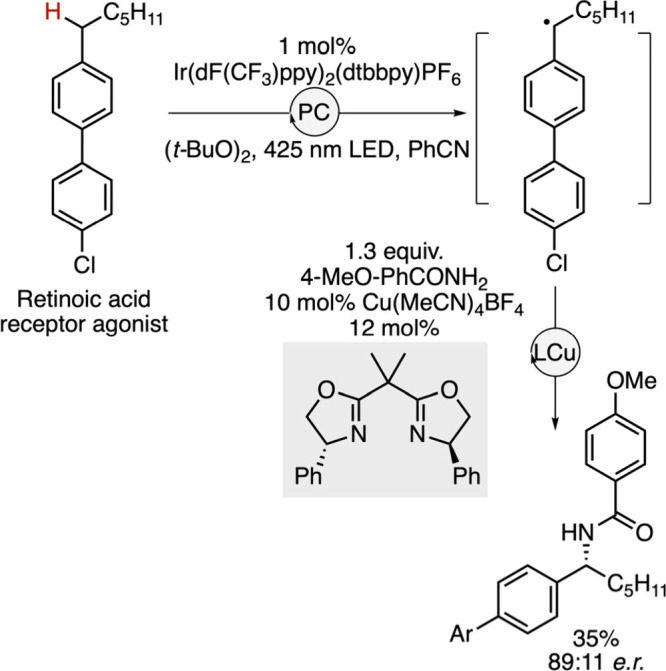
Catalytic Enantioselective Late-Stage C–H Radical Amidation

The last five years have witnessed the emergence of a new concept in synthesis, i.e., the so-called “molecular editing” that can be considered as a chemical site-directed mutagenesis.^128^ In a single step, it is possible to modify the core of complex products according to several elementary operations that include rearrangements (ring expansion or contraction), mutations (deletion or insertion of a single atom) or transmutations (replacement of one atom by another one).^128^ Needless to mention, these transformations raise new challenging issues in asymmetric catalysis that remain to be addressed. In this context, Bi and co-workers very recently reported a first breakthrough with the dirhodium­(II)-catalyzed enantioselective homologation of indoles (Scheme 26).^143^ The reaction proceeds through the initial nucleophilic attack of the indole on the chiral rhodium-bound carbene generated from a specific trifluoromethylhydrazone. This is the enantiodetermining step of the process that is followed by the formation of a cyclopropane and its subsequent NaH-mediated rearrangement to the 3,4-dihydroquinoline derivative. This last step occurs with retention of configuration to deliver an optically pure CF_3_-containing compound of utmost interest in medicinal chemistry. Interestingly, this process can also be applied to the homologation of pyrroles. Moreover, the clever design of new rhodium-bound carbyne species generated from a leaving group-containing diazo compound enabled the development of one of the first atroposelective single-carbon insertion into indoles for the formation of atropochiral quinolines.^144^

**Scheme 26:**
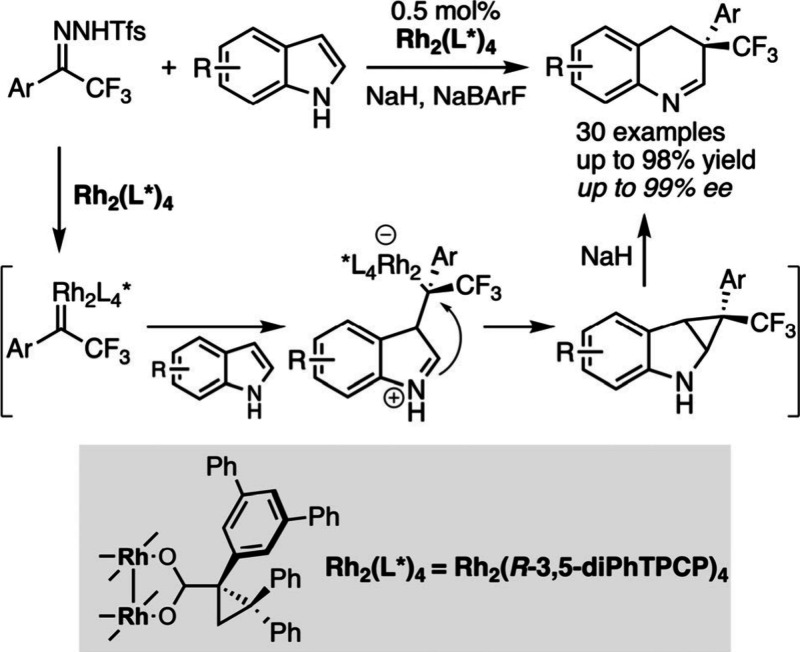
Catalytic Enantioselective Homologation of Indoles

The search for innovation in synthesis still challenges the creativity of organic chemists who manage to address issues of increasing complexity. New reagents and catalysts as well as relevant conditions are developed to this end with the aim to report efficient selective processes. However, these major achievements are often accomplished without taking sustainability criteria into account. This includes high catalyst loading to reach good conversion, the design of nonreadily available complex ligand, or the use of irrelevant solvents. The rise of C–H bond activation associated with the use of 1,2-dichloroethane is a textbook case in this context, which required to look for alternative solvents to develop a sustainable chemistry.^145^

## 6 Conclusion

In conclusion, asymmetric catalysis is a central science to sustainable organic synthesis because of the ever-increasing need for chiral enantioenriched molecules that are essential to our society. As described in this Outlook, various innovative strategies have recently been implemented to develop enantioselective reactions incorporating more benign or earth abundant catalysts together with milder and greener reaction conditions. In addition, the scope of substrates amenable to enantioselective transformations has also considerably expanded over the last decades. Now, it encompasses a range of readily available unactivated substrates derived from biomass and renewable resource, thus enabling synthetic chemists to explore an always-broader chiral chemical space.

We have shown in this Outlook which new strategies have been developed recently, but we do not aim to specify which ones are the most promising to pursue. Synthesis chemists continue to demonstrate creativity in many different areas but they are nowadays fully aware of the need to embrace sustainability. This was not necessarily required in the past when developing high value-added products, but those days are over. A current challenge for asymmetric catalysis is the identification of metrics to assess the sustainability of a given process, in particular when sophisticated catalytic systems are involved. While process mass intensity or its derived E-factor were traditionally selected by the industry,^146,147^ the focus has recently shifted toward life cycle assessment that enables an holistic sustainability evaluation.^148,149^ However, this approach is data intensive and is currently hindered by the limited availability of production data for fine chemicals.

Obviously, asymmetric catalysis must face other challenges than sustainability. Indeed, creativity still must be at the forefront in order to tackle new challenges in this area such as the selective synthesis of stereogenic atoms other than carbon as well as axially-, helically- or planar-chiral structures. In addition, asymmetric catalysis could also be involved in solving a fundamental but yet elusive question that is the origin of biological homochirality on Earth.^150^ To face all these challenges, synthetic chemists will probably rely more and more on novel enabling technologies such as high-throughput experimentation and the use of artificial intelligence.

## Notes

This Outlook is dedicated to Professor Henri B. Kagan for his pioneering and outstanding contribution in the area of asymmetric catalysis.

## References

[1] Brooks H. W., Guida C. W., Daniel G. K. (2011). The significance of chirality in
drug design and development. Curr. Top. Med.
Chem..

[2] McVicker R. U., O’Boyle N. M. (2024). Chirality of New Drug Approvals (2013–2022):
Trends and Perspectives. J. Med. Chem..

[3] Jeschke P. (2025). The continuing
significance of chiral agrochemicals. Pest.
Manag. Sci..

[4] Worch J. C., Prydderch H., Jimaja S., Bexis P., Becker M. L., Dove A. P. (2019). Stereochemical
enhancement of polymer properties. Nat. Rev.
Chem..

[5] Brandt J. R., Salerno F., Fuchter M. J. (2017). The added value of small-molecule
chirality in technological applications. Nat.
Rev. Chem..

[6] Nozaki H., Moriuti S., Takaya H., Noyori R. (1966). Asymmetric induction
in carbenoid reaction by means of a dissymmetric copper chelate. Tetrahedron Lett..

[7] Knowles W. S., Sabacky M. J. (1968). Catalytic asymmetric hydrogenation
employing a soluble,
optically active, rhodium complex. J. Chem.
Soc. Chem. Commun..

[8] Dang T. P., Kagan H. B. (1971). The asymmetric synthesis
of hydratopic acid and amino-acids
by homogeneous catalytic hydrogenation. J. Chem.
Soc. Chem. Commun..

[9] Katsuki T., Sharpless K. B. (1980). The first
practical method for asymmetric epoxidation. J. Am. Chem. Soc..

[10] Knowles W. S. (2002). Asymmetric
hydrogenations. Angew. Chem., Int. Ed..

[11] Noyori R. (2002). Asymmetric
catalysis: science and opportunities. Angew.
Chem., Int. Ed..

[12] Sharpless K. B. (2002). Searching
for new reactivity. Angew. Chem., Int. Ed..

[13] Pracejus H. (1960). Asymmetrische
Synthesen mit Ketenen, I. Alkaloid-katalysierte asymmetrische Synthesen
von α-Phenyl-propionsäureestern. Justus Liebigs Ann.Chem..

[14] Eder U., Sauer G., Wiechert R. (1971). New type of asymmetric
cyclization
to optically active steroid CD partial structures. Angew. Chem., Int. Ed..

[15] Hajos Z. G., Parrish D. R. (1974). Asymmetric synthesis of bicyclic intermediates of natural
product chemistry. J. org. Chem..

[16] Dalko P. I., Moisan L. (2004). In the golden age of
organocatalysis. Angew. Chem., Int. Ed..

[17] List B., Lerner R. A., Barbas C. F. (2000). Proline-catalyzed
direct asymmetric
aldol reactions. J. Am. Chem. Soc..

[18] Ahrendt K. A., Borths C. J., MacMillan D. C. W. (2000). New
strategies for organic catalysis:
the first highly enantioselective organocatalytic Diels-Alder reaction. J. Am. Chem. Soc..

[19] Rosenthaler L. (1908). Durch Enzyme
bewirkte asymmetrische Synthese. Biochem. Z..

[20] Bornscheuer U. T., Huisman G. W., Kazlausks R. J., Lutz S., Moore J. C., Robins K. (2012). Engineering
the third
wave of biocatalysis. Nature.

[21] Anastas, P. T. ; Warner, J. C. Green Chemistry: Theory and Practice; Oxford University Press: Oxford, UK, 1998. 10.1093/oso/9780198506980.001.0001.

[22] Zhang Z., Zhu G., Jiang Q., Xiao D., Zhang X. (1999). Highly Enantioselective Hydrogenation
of Cyclic Enamides Catalyzed by a Rh-PennPhos Catalyst. J. Org. Chem..

[23] Knowles W. S. (1983). Asymmetric
hydrogenation. Acc. Chem. Res..

[24] Aratani T. (1985). Catalytic
Asymmetric Synthesis of Cyclopropanecarboxylic Acids: An Application
of Chiral Copper Carbenoid Reaction. Pure. Appl.
Chem..

[25] Dumont W., Poulin J. C., Phat D. T., Kagan H. B. (1973). Asymmetric Catalytic
Reduction with TransitionMetal Complexes. II. Asymmetric Catalysis
by aSupported Chiral Rhodium Complex. J. Am.
Chem. Soc..

[26] Takaishi N., Imai H., Bertelo C. A., Stille J. K. (1976). Transition
Metal Catalyzed Asymmetric Organic Syntheses
via Polymer Bound Chiral Ligands. Synthesis of R Amino Acids and Flydratropic
Acid by Hydrogenation. J. Am. Chem. Soc..

[27] Atodiresei I., Vila C., Rueping M. (2015). Asymmetric Organocatalysis in Continuous
Flow: Opportunities for Impacting Industrial Catalysis. ACS Catal..

[28] Xu H., Gao J., Jiang D. (2015). Stable, crystalline, porous, covalent
organic frameworks
as a platform for chiral organocatalysts. Nat.
Chem..

[29] Bae H. Y., Hofler D., Kaib P. S. J., Kasaplar P., De C. K., Dohring A., Lee S., Kaupmees K., Leito I., List B. (2018). Approaching sub-ppm-level
asymmetric organocatalysis of a highly
challenging and scalable carbon–carbon bond forming reaction. Nat. Chem..

[30] Antenucci A., Dughera S., Renzi P. (2021). Green Chemistry Meets
Asymmetric
Organocatalysis: A Critical Overview on Catalysts Synthesis. ChemSusChem.

[31] Han B., He X.-H., Liu Y.-Q., He G., Peng C., Li J.-L. (2021). Asymmetric organocatalysis: an enabling technology for medicinal
chemistry. Chem. Soc. Rev..

[32] Rechavi D., Lemaire M. (2002). Enantioselective Catalysis
Using Heterogeneous Bis­(oxazoline) Ligands: Which Factors Influence
the Enantioselectivity?. Chem. Rev..

[33] Ren Y., Wang M., Yang Q., Zhu J. (2023). Merging Chiral Diamine
and Ni/SiO2 for Heterogeneous Asymmetric 1,4-Addition Reactions. ACS Catal..

[34] Hübner S., de Vries J. G., Farina V. (2016). Why Dos Industry Not
Use Immobilized
Transition Metal Complexes as Catalysts?. Adv.
Synth. Catal..

[35] Huang X.-Y., Zheng Q., Zou L.-M., Gu Q., Tu T., You S.-L. (2022). Hyper-Crosslinked Porous Chiral Phosphoric Acids: Robust
Solid Organocatalysts for Asymmetric Dearomatization Reactions. ACS Catal..

[36] Zheng W., Tan R., Yin S., Zhang Y., Zhao G., Chen Y., Yin D. (2015). Ionic liquid-functionalized
graphene oxide as an efficient support
for the chiral salen M­(III) complex in asymmetric epoxidation of unfunctionalized
olefins. Catal. Sci. Technol..

[37] Xing C., Deng J., Tan R., Gao M., Hao P., Yin D., Yin D. (2017). Cooperative chiral
salen Ti­(IV) catalyst supported
on ionic liquid-functionalized graphene oxide accelerates asymmetric
sulfoxidation in water. Catal. Sci. Technol..

[38] Jacobsen E.
N. (2000). Asymmetric Catalysis
of Epoxide Ring-Opening
Reactions. Acc. Chem. Res..

[39] Belser T., Jacobsen E. N. (2008). Cooperative Catalysis
in the Hydrolytic Kinetic Resolution
of Epoxides by Chiral [(salen)­Co­(III)] Complexes Immobilized on Gold
Colloids. Adv. Synth. Catal..

[40] Hong X., Mellah M., Bordier F., Guillot R., Schulz E. (2012). Electrogenerated
Polymers as Efficient and Robust Heterogeneous Catalysts for the Hydrolytic
Kinetic Resolution of Terminal Epoxides. ChemCatChem..

[41] Egorova K. S., Ananikov V. P. (2016). Which Metals are
Green for Catalysis? Comparison of
the Toxicities of Ni, Cu, Fe, Pd, Pt, Rh, and Au Salts. Angew. Chem., Int. Ed..

[42] Pellissier H. (2019). Green Copper
Catalysis in Enantioselective Domino Reactions. Curr. Org. Chem..

[43] Zhu S., Niljianskul N., Buchwald S. L. (2013). Enantio- and Regioselective CuH-Catalyzed
Hydroamination of Alkenes. J. Am. Chem. Soc..

[44] Miki Y., Hirano K., Satoh T., Miura M. (2013). Copper-Catalyzed Intermolecular
Regioselective Hydroamination of Styrenes with Polymethylhydrosiloxane
and Hydroxylamines. Angew. Chem., Int. Ed..

[45] Junge K., Wendt B., Addis D., Zhou S., Das S., Fleischer S., Beller M. (2011). Copper-Catalyzed Enantioselective
Hydrogenation of Ketones. Chem.—Eur.
J..

[46] Zatolochnaya O. V., Rodríguez S., Zhang Y., Lao K. S., Tcyrulnikov S., Li G., Wang X.-J., Qu B., Biswas S., Mangunuru H. P. R., Rivalti D., Sieber J. D., Desrosiers J.-N., Leung J. C., Grinberg N., Lee H., Haddad N., Yee N. K., Song J. J., Kozlowski M. C., Senanayake C. H. (2018). Copper-catalyzed asymmetric hydrogenation of 2-substituted
ketones via dynamic kinetic resolution. Chem.
Sci..

[47] Zheng Y., Wang E. H. N., Rizzo A., Chiu P. (2025). Enantiomerically Enriched
Aziridine-2-carboxylates via Copper-Catalyzed Reductive Kinetic Resolution
of 2H-Azirines. Angew. Chem., Int. Ed..

[48] Pellissier H. (2024). Enantioselective
iron-catalysed transformations. An update. Tetrahedron.

[49] Feng S.-X., Lu Q.-T., Lu Y., Cui L., Yang L., Baghel A. S., Cai Q. (2025). Iron-Catalyzed Asymmetric
[4 + 2]/Cheletropic
Retro-[4 + 1] Cycloadditions of Thiophene S,S-Dioxides with 3-Substituted
Indoles. Angew. Chem., Int. Ed..

[50] Morris R. H. (2015). Exploiting
Metal–Ligand Bifunctional Reactions in the Design of Iron Asymmetric
Hydrogenation Catalysts. Acc. Chem. Res..

[51] Hong Y., Jarrige L., Harms K., Meggers E. (2019). Chiral-at-Iron Catalyst:
Expanding the Chemical Space for Asymmetric Earth-Abundant Metal Catalysis. J. Am. Chem. Soc..

[52] Wang C., An B., Lin W. (2019). Metal-Organic Frameworks in Solid-Gas Phase Catalysis. ACS Catal..

[53] Wu C.-D, Hu A., Zhang L., Lin W. (2005). A Homochiral
Porous Metal-Organic
Framework for Highly Enantioselective Heterogeneous Asymmetric Catalysis. J. Am. Chem. Soc..

[54] Gong W., Chen Z., Dong J., Liu Y., Cui Y. (2022). Chiral Metal–Organic
Frameworks. Chem. Rev..

[55] Xia Q., Liu Y., Li Z., Gong W., Cui Y. (2016). A Cr­(salen)-based metal–organic
framework as a versatile catalyst for efficient asymmetric transformations. Chem. Commun..

[56] Fan Y., Ren Y., Li J., Yue C. H. (2018). Enhanced Activity and Enantioselectivity
of Henry Reaction by the Postsynthetic Reduction Modification for
a Chiral Cu­(salen)-Based Metal–Organic Framework. Inorg. Chem..

[57] Zhu C., Xia Q., Chen X., Liu Y., Du X., Cui Y. (2016). Chiral Metal–Organic
Framework as a Platform for Cooperative Catalysis in Asymmetric Cyanosilylation
of Aldehydes. ACS Catal..

[58] Tan C., Han X., Li Z., Liu Y., Cui Y. (2018). Controlled Exchange
of Achiral Linkers with Chiral Linkers in Zr-Based UiO-68 Metal–Organic
Framework. J. Am. Chem. Soc..

[59] Jiang H., Zhao X., Zhang W., Liu Y., Li H., Cui Y. (2023). Conformational Control of Organocatalyst
in Strongly Brønsted
Acidic Metal-Organic Frameworks for Enantioselective Catalysis. Angew. Chem., Int. Ed..

[60] Guo J., Jiang D. (2020). Covalent Organic Frameworks for Heterogeneous Catalysis: Principle,
Current Status, and Challenges. ACS Cent. Sci..

[61] Han X., Xia Q., Huang J., Liu Y., Tan C., Cui Y. (2017). Chiral Covalent
Organic Frameworks with High Chemical Stability for Heterogeneous
Asymmetric Catalysis. J. Am. Chem. Soc..

[62] Yuan C., Fu S., Kang X., Cheng C., Jiang C., Liu Y., Cui Y. (2024). Mixed-Linker
Chiral 2D Covalent Organic Frameworks with Controlled
Layer Stacking for Electrochemical Asymmetric Catalysis. J. Am. Chem. Soc..

[63] Staveness D., Bosque I., Stephenson C. R. J. (2016). Free
Radical Chemistry Enabled by Visible Light-Induced Electron Transfer. Acc. Chem. Res..

[64] Wills A. G., Charvet S., Battilocchio C., Scarborough C. C., Wheelhouse K. M. P., Poole D. L., Carson N., Vantourout J. C. (2021). High-Throughput
Electrochemistry: State of the Art, Challenges, and Perspective. Org. Process Res. Dev..

[65] Yan M., Kawamata Y., Baran P. S. (2018). Synthetic Organic Electrochemistry:
Calling All Engineers. Angew. Chem., Int. Ed..

[66] Horn E. J., Rosen B. R., Baran P. S. (2016). Synthetic
organic electrochemistry:
an enabling and innately sustainable method. ACS Cent. Sci..

[67] John
David A., Balaji R., Das A. (2025). Adv. Advances of Chiral
Metal Complexes as Standalone Photocatalyst for Asymmetric Organic
Transformations. Synth. Catal..

[68] Meggers E. (2011). Asymmetric
Synthesis of Octahedral Coordination Complexes. Eur. J. Inorg. Chem..

[69] Tan Y., Yuan W., Gong L., Meggers E. (2015). Aerobic Asymmetric Dehydrogenative Cross-Coupling between
Two Csp3-H Groups Catalyzed by a Chiral-at-Metal Rhodium Complex. Angew. Chem..

[70] Huo H., Shen X., Wang C., Zhang L., Röse P., Chen L. A., Harms K., Marsch M., Hilt G., Meggers E. (2014). Asymmetric photoredox
transition-metal catalysis activated
by visible light. Nature.

[71] Tlili A., Lakhdar S. (2021). Acridinium Salts and
Cyanoarenes as Powerful Photocatalysts:
Opportunities in Organic Synthesis. Angew. Chem.,
Int. Ed..

[72] Paria S., Reiser O. (2014). Copper in Photocatalysis. ChemCatChem..

[73] Woodhouse M. D., McCusker J. K. (2020). Mechanistic Origin of Photoredox Catalysis Involving
Iron­(II) Polypyridyl Chromophores. J. Am. Chem.
Soc..

[74] Wang P.-Z., Chen J.-R., Xia W.-J. (2019). Hantzsch esters: an emerging versatile
class of reagents in photoredox catalyzed organic synthesis. Org. Biomol. Chem..

[75] Han T., Mou Q., Lv Y., Liu M. (2024). Light-driven asymmetric coupling
of aromatic aldehydes and aryl iodides using a simple amine reductant. Green Chem..

[76] Yan M., Kawamata Y., Baran P. S. (2017). Synthetic Organic Electrochemical
Methods Since 2000: On the Verge of a Renaissance. Chem. Rev..

[77] Gourley R. N., Grimshaw J., Millar P. G. (1967). Electrochemical
reduction in the
presence of tertiary amines: an asymmetric synthesis of 3, 4-Dihydro-4-methylcoumarin. Chem. Commun..

[78] Durandetti M., Périchon J., Nédélec J. Y. (1997). Asymmetric induction
in the electrochemical cross-coupling of aryl halides with a-chloropropionic
acid derivatives catalyzed by nickel complexes. J. Org. Chem..

[79] Amundsen A. R., Balko E. N. (1992). Preparation of chiral diols by the osmiumcatalysed,
indirect anodic oxidation of olefins. J. Appl.
Electrochem..

[80] Torii S., Liu P., Tanaka H. (1995). Electrochemical
Os-catalyzed asymmetric dihydroxylation of olefins with sharpless’
ligand. Chem. Lett..

[81] Tanaka H., Kuroboshi M., Takeda H., Kanda H., Torii S. (2001). Electrochemical
asymmetric epoxidation of olefins by using an optically active Mn-salen
complex. J. Electroanal. Chem..

[82] Fu N., Song L., Liu J., Shen Y., Siu J. C., Lin S. (2019). New Bisoxazoline Ligands
Enable Enantioselective Electrocatalytic
Cyanofunctionalization of Vinylarenes. J. Am.
Chem. Soc..

[83] Chen J., Teng M., Huang F., Song H., Wang Z., Zhuang H., Wu Y., Wu X., Yao Q., Shi B. (2022). Cobalt/Salox-Catalyzed Enantioselective Dehydrogenative C–H
Alkoxylation and Amination. Angew. Chem., Int.
Ed..

[84] Zhou G., Chen J., Yao Q., Huang F., Wang Z., Shi B. (2023). Base-Promoted Electrochemical
Co­(II) -catalyzed Enantioselective
C–H Oxygenation. Angew. Chem. Int. Ed.

[85] Meyer T. H., Oliviera J. C. A., Ghorai D. G., Ackermann L. (2020). Insights into
Cobalta­(III/IV/II)-Electrocatalysis: Oxidation-Induced Reductive Elimination
for Twofold C–H Activation. Angew. Chem.,
Int. Ed..

[86] Von
Münchow T., Dana S., Xu Y., Yuan B., Ackermann L. (2023). Enantioselective electrochemical cobalt-catalyzed aryl
C–H activation reactions. Science.

[87] Cherney A. H., Reisman S. E. (2014). Nickel-Catalyzed
Asymmetric Reductive Cross-Coupling
Between Vinyl and Benzyl Electrophiles. J. Am.
Chem. Soc..

[88] DeLano T.
J., Reisman S. E. (2019). Enantioselective
Electroreductive Coupling of Alkenyl
and Benzyl Halides via Nickel Catalysis. ACS
Catal..

[89] Qiu H., Shuai B., Wang Y.-Z., Liu D., Chen Y.-G., Gao P.-S., Ma H.-X., Chen S., Mei T.-S. (2020). Enantioselective
Ni-Catalyzed Electrochemical Synthesis of Biaryl Atropisomers. J. Am. Chem. Soc..

[90] Hu X., Cheng-Sánchez I., Cuesta-Galisteo S., Nevado C. (2023). Nickel-Catalyzed Enantioselective
Electrochemical Reductive Cross-Coupling of Aryl Aziridines with Alkenyl
Bromides. J. Am. Chem. Soc..

[91] Tao Y., Ma W., Sun R., Huang C., Lu Q. (2024). Asymmetric Paired Electrolysis:
Enantioselective Alkylation of Sulfonylimines via C­(Sp3)–H
Functionalization. Angew. Chem., Int. Ed..

[92] Zhao D., Ding K. (2013). Recent Advances in
Asymmetric Catalysis in Flow. ACS Catal..

[93] Saito Y., Sato Y., Kobayashi S. (2024). Continuous-Flow
Enantioselective
Hydrogenative Enyne Cyclization with Chiral Heterogeneous Rh Catalysts. ACS Catal..

[94] Zhou Z., Kasten K., Kang T., Cordes D. B., Smith A. D. (2024). Enantioselective
Synthesis in Continuous Flow: Polymer-Supported Isothiourea-Catalyzed
Enantioselective Michael Addition–Cyclization with α-Azol-2-ylacetophenones. Org. Process Res. Dev..

[95] Chen P.-Y., Huang C., Jie L.-H., Guo B., Zhu S., Xu H.-C. (2024). Unlocking the Potential of Oxidative Asymmetric Catalysis
with Continuous
Flow Electrochemistry. J. Am. Chem. Soc..

[96] Rodríguez B., Rantanen T., Bolm C. (2006). Solvent-Free
Asymmetric Organocatalysis
in a Ball Mill. Angew. Chem., Int. Ed..

[97] Némethová V., Krištofiková D., Mečiarová M., Šebesta R. (2023). Asymmetric
Organocatalysis Under Mechanochemical Conditions. Chem. Rec..

[98] Staleva P., Hernandez J. G., Bolm C. (2019). Mechanochemical Copper-Catalyzed
Asymmetric Michael-Type Friedel–Crafts Alkylation of Indoles
with Arylidene Malonates. Chem.—Eur.
J..

[99] Bolt R. R. A., Raby-Buck S. E., Ingram K., Leitch J. A., Browne D. L. (2022). Temperature-Controlled
Mechanochemistry for the Nickel-Catalyzed Suzuki–Miyaura-Type
Coupling of Aryl Sulfamates via Ball Milling and Twin-Screw Extrusion. Angew. Chem., Int. Ed..

[100] Gonnet L., Lennox C. B., Do J.-L., Malvestiti I., Koenig S. G., Nagapudi K., Friščić T. (2022). Metal-Catalyzed
Organic Reactions by Resonant Acoustic Mixing. Angew. Chem., Int. Ed..

[101] Martínez S., Veth L., Lainer B., Dydio P. (2021). Challenges
and Opportunities in Multicatalysis. ACS Catal..

[102] Ambrosini L. M., Lambert T. H. (2010). Multicatalysis:
Advancing Synthetic
Efficiency and Inspiring Discovery. ChemCatChem..

[103] Krautwald S., Sarlah D., Schafroth M. A., Carreira E. M. (2013). Enantio- and Diastereodivergent Dual Catalysis: α-Allylation
of Branched Aldehydes. Science.

[104] Huffman M. A., Fryszkowska A., Alvizo O., Bor-ra-Garske M., Campos K. R., Canada K. A., Devine P. N., Duan D., Forstater J. H., Grosser S. T., Halsey H. M., Hughes G. J., Jo J., Joyce L. A., Kolev J. N., Liang J., Maloney K. M., Mann B. F., Marshall N. M., McLaughlin M., Moore J. C., Murphy G. S., Nawrat C. C., Nazor J., Novick S., Patel N. R., Rodriguez-Granillo A., Robaire S. A., Sherer E. C., Truppo M. D., Whittaker A. M., Ver-ma D., Xiao L., Xu Y., Yang H. (2019). Design of
an in Vitro Biocatalytic Cascade for the Manufacture of Islatravir. Science.

[105] Reed-Berendt B. G., Latham D. E., Dambatta M. B., Morrill L. C. (2021). Borrowing
Hydrogen for Organic Synthesis. ACS Cent. Sci..

[106] Quintard A., Constantieux T., Rodriguez J. (2013). An Iron/Amine-Catalyzed
Cascade Process for the Enantioselective Functionalization of Allylic
Alcohols. Angew. Chem., Int. Ed..

[107] Quintard A., Rodriguez J. (2014). Iron Cyclopentadienone
Complexes:
Discovery, Properties, and Catalytic Reactivity. Angew. Chem., Int. Ed..

[108] Palomo C., Mielgo A. (2006). Diarylprolinol Ethers: Expanding
the Potential of Enamine/Iminium-Ion Catalysis. Angew. Chem., Int. Ed..

[109] Chang X., Cheng X., Liu X.-T., Fu C., Wang W.-Y., Wang C.-J. (2022). Stereodivergent Construction of 1,4-Nonadjacent
Stereocenters via Hydroalkylation of Racemic Allylic Alcohols Enabled
by Copper/Ruthenium Relay Catalysis. Angew.
Chem., Int. Ed..

[110] Allen A. E., MacMillan D. W. C. (2012). Synergistic Catalysis: A Powerful
Synthetic Strategy for New Reaction Development. Chem. Sci..

[111] Zhou S., Fleischer S., Junge K., Beller M. (2011). Cooperative
Transition-Metal and Chiral Brønsted Acid Catalysis: Enantioselective
Hydrogenation of Imines To Form Amines. Angew.
Chem., Int. Ed..

[112] Johnson N. B., Lennon I. C., Moran P. H., Ramsden J. A. (2007). Industrial-Scale
Synthesis and Applications of Asymmetric Hydrogenation Catalysts. Acc. Chem. Res..

[113] Chirik P. J. (2015). Iron- and
Cobalt-Catalyzed Alkene Hydrogenation: Catalysis
with Both Redox-Active and Strong Field Ligands. Acc. Chem. Res..

[114] Hong B.-C. (2020). Enantioselective Synthesis Enabled by Visible Light
Photocatalysis. Org. Biomol. Chem..

[115] Proctor R. S. J., Davis H. J., Phipps R. J. (2018). Catalytic
Enantioselective
Minisci-Type Addition to Heteroarenes. Science.

[116] Bacoş P. D., Lahdenperä A.
S. K., Phipps R. J. (2023). Discovery
and Development of the Enantioselective Minisci Reaction. Acc. Chem. Res..

[117] Marshall C. M., Federice J. G., Bell C. N., Cox P. B., Njardarson J. T. (2024). An Update on the Nitrogen Heterocycle Compositions
and Properties of U.S. FDA-Approved Pharmaceuticals (2013–2023). J. Med. Chem..

[118] Liu X., Liu Y., Chai G., Qiao B., Zhao X., Jiang Z. (2018). Organocatalytic Enantioselective
Addition of α-Aminoalkyl Radicals
to Isoquinolines. Org. Lett..

[119] Fu M.-C., Shang R., Zhao B., Wang B., Fu Y. (2019). Photocatalytic Decarboxylative Alkylations
Mediated by Triphenylphosphine
and Sodium Iodide. Science.

[120] Wang P.-Z., Zhang B., Xiao W.-J., Chen J.-R. (2024). Photocatalysis
Meets Copper Catalysis: A New Opportunity for Asymmetric Multicomponent
Radical Cross-Coupling Reactions. Acc. Chem.
Res..

[121] Xu W., XU T. (2024). Dual Nickel-
and Photoredox-Catalyzed Asymmetric Reductive
Cross-Couplings: Just a Change of the Reduction System?. Acc. Chem. Res..

[122] Xu Y., Lin Y., Homölle S. L., Oliveira J. C. A., Ackermann L. (2024). Enantioselective
Cobaltaphotoredox-Catalyzed C–H Activation. J. Am. Chem. Soc..

[123] Chen X., Kramer S. (2024). Photoinduced Transition-Metal-Catalyzed
Enantioselective Functionalization of Non-Acidic C­(sp^3^)–H
Bonds. Chem. Catal..

[124] Großkopf J., Bach T. (2023). Catalytic Photochemical
Deracemization
via Short-Lived Intermediates. Angew. Chem.,
Int. Ed..

[125] Du J., Skubi K. L., Schultz D. M., Yoon T. P. (2014). A Dual-Catalysis
Approach to Enantioselective [2 + 2] Photocycloadditions Using Visible
Light. Science.

[126] Sun A. W., Lackner S., Stoltz B. M. (2019). Modularity:
adding
new dimensions to total synthesis. Trends Chem..

[127] Castellino N. J., Montgomery A. P., Danon J. J., Kassiou M. (2023). Late-stage
functionalization for improving drug-like molecular properties. Chem. Rev..

[128] Jurczyk J., Woo J., Kim S. F., Dherange B. D., Sarpong R., Levin M. D. (2022). Single-atom logic for heterocycle
editing. Nat. Synth..

[129] Huang G., Kouklovsky C., de la Torre A. (2022). Gram-scale
enantioselective synthesis of (+)-Lucidumone. J. Am. Chem. Soc..

[130] Huang G., Guillot R., Kouklovsky C., Maryasin B., de la Torre B. (2022). Diastereo- and enantioselective inverse-electron-demand
Diels-Alder cycloaddition between 2-pyrones and acyclic enol ethers. Angew. Chem., Int. Ed..

[131] Godula K., Sames D. (2006). C–H Bond Functionalization
in Complex Organic Synthesis. Science.

[132] Stout C. N., Wasfy N. M., Chen F., Renata H. (2023). Charting the Evolution of Chemoenzymatic Strategies
in the Synthesis of Complex Natural Products. J. Am. Chem. Soc..

[133] Saint-Denis T. G., Zhu R.-Y., Chen G., Wu Q.-F., Yu J.-Q. (2018). Enantioselective C­(sp^3^)–H bond activation by chiral
transition metal catalysts. Science.

[134] Guo S.-M., Huh S., Coelho M., Shen L., Peters G., Baudoin O. (2023). A C–H activation-based
enantioselective
synthesis of lower carbo­[*n*]­helicenes. Nat. Chem..

[135] Huh S., Linne E., Estaque L., Peters G., Devereux M., Baudoin O. (2025). A C–H arylation-based enantioselective
synthesis
of planar chiral cyclophanes. Angew. Chem.,
Int. Ed..

[136] Chu J. C. K., Rovis T. (2018). Complementary Strategies for Directed
C­(sp^3^)–H Functionalization: A Comparison of Transition-Metal-Catalyzed
Activation, Hydrogen Atom Transfer, and Carbene/Nitrene Transfer. Angew. Chem., Int. Ed..

[137] Davies H. M. L., Liao K. (2019). Dirhodium Tetracarboxylates as Catalysts
for Selective Intermolecular C–H Functionalization. Nat. Rev. Chem..

[138] Garlets Z. J., Sanders J. N., Malik H., Gampe C., Houk K. N., Davies H. M. L. (2020). Enantioselective C–H functionalization
of bicyclo[1.1.1]­pentanes. Nat. Catal..

[139] Brunard E., Boquet V., Saget T., Sosa Carrizo E. D., Sircoglou M., Dauban P. (2024). Catalytic-controlled
Intermolecular
homobenzylic C­(sp^3^)–H amination for the synthesis
of β-arylethylamines. J. Am. Chem. Soc..

[140] Nasrallah A., Boquet V., Hecker A., Retailleau P., Darses B., Dauban P. (2019). Catalytic Enantioselective
Intermolecular
Benzylic C­(sp^3^)–H Amination. Angew. Chem., Int. Ed..

[141] Capaldo L., Ravelli D., Fagnoni M. (2022). Direct photocatalyzed
hydrogen atom transfer (HAT) for aliphatic C–H bonds elaboration. Chem. Rev..

[142] Chen X., Lian Z., Kramer S. (2023). Enantioselective intermolecular
radical amidation and amination of benzylic C–H bonds via dual
copper and photocatalysis. Angew. Chem., Int.
Ed..

[143] Zhang X., Song Q., Liu S., Sivaguru P., Liu Z., Yang Y., Ning Y., Anderson E. A., de Ruiter G., Bi X. (2025). Asymmetric dearomative
single-atom skeletal editing of indoles and
pyrroles. Nat. Chem..

[144] Li B., Alfonso V. G., Puggioli A., Solé-Daura A., Maseras F., Suero M. G. (2025). Rh-Catalyzed atroposelective
single-carbon
edition. J. Am. Chem. Soc..

[145] Sherwood J. (2018). European Restrictions on 1,2-Dichloroethane:
C–H
Activation Research and Development Should Be liberated and not Limited. Angew. Chem. Int. Ed..

[146] Jimenez-Gonzalez C., Ponder C. S., Broxterman Q. B., Manley J. B. (2011). Using the Right Green Yardstick: Why Process Mass Intensity
Is Used in the Pharmaceutical Industry To Drive More Sustainable Processes. Org. Process Res. Dev..

[147] Jiménez-González C., Ollech C., Pyrz W., Hughes D., Broxterman Q. B., Bhathela N. (2013). Expanding the Boundaries:
Developing a Streamlined Tool for Eco-Footprinting of Pharmaceuticals. Org. Process Res. Dev..

[148] Sheldon R. A. (2018). Metrics
of Green Chemistry and Sustainability: Past,
Present, and Future. ACS Sustainable Chem. Eng..

[149] Folkerts S., Hoepfner M. G., Guillén-Gosálbez G., Pérez-Ramírez J., Carreira E. (2025). Integrated Life Cycle
Assessment Guides Sustainability in Synthesis: Antiviral Letermovir
as a Case Study. J. Am. Chem. Soc..

[150] Blackmond D. G. (2004). Asymmetric Autocatalysis and Its
Implications for the
Origin of Homochirality. Proc. Nat. Ac. Sci..

